# Tetraspanin Is Required for Generation of Reactive Oxygen Species by the Dual Oxidase System in *Caenorhabditis elegans*

**DOI:** 10.1371/journal.pgen.1002957

**Published:** 2012-09-20

**Authors:** Hiroki Moribe, Ryouji Konakawa, Daisuke Koga, Tatsuo Ushiki, Kuniaki Nakamura, Eisuke Mekada

**Affiliations:** 1 Department of Biology, Kurume University School of Medicine, Kurume, Fukuoka, Japan; 2 Department of Cell Biology, Research Institute for Microbial Diseases, Osaka University, Suita, Osaka, Japan; 3 Division of Microscopic Anatomy and Bio-Imaging, Niigata University Graduate School of Medical and Dental Sciences, Chuo-ku, Niigata, Japan; University of California San Diego, United States of America

## Abstract

Reactive oxygen species (ROS) are toxic but essential molecules responsible for host defense and cellular signaling. Conserved NADPH oxidase (NOX) family enzymes direct the regulated production of ROS. Hydrogen peroxide (H_2_O_2_) generated by dual oxidases (DUOXs), a member of the NOX family, is crucial for innate mucosal immunity. In addition, H_2_O_2_ is required for cellular signaling mediated by protein modifications, such as the thyroid hormone biosynthetic pathway in mammals. In contrast to other NOX isozymes, the regulatory mechanisms of DUOX activity are less understood. Using *Caenorhabditis elegans* as a model, we demonstrate that the tetraspanin protein is required for induction of the DUOX signaling pathway in conjunction with the dual oxidase maturation factor (DUOXA). In the current study, we show that genetic mutation of DUOX *(bli-3)*, DUOXA *(doxa-1)*, and peroxidase (*mlt-7*) in *C. elegans* causes the same defects as a tetraspanin *tsp-15* mutant, represented by exoskeletal deficiencies due to the failure of tyrosine cross-linking of collagen. The deficiency in the *tsp-15* mutant was restored by co-expression of *bli-3* and *doxa-1*, indicating the involvement of *tsp-15* in the generation of ROS. H_2_O_2_ generation by BLI-3 was completely dependent on TSP-15 when reconstituted in mammalian cells. We also demonstrated that TSP-15, BLI-3, and DOXA-1 form complexes *in vitro* and *in vivo*. Cell-fusion-based analysis suggested that association with TSP-15 at the cell surface is crucial for BLI-3 activation to release H_2_O_2_. This study provides the first evidence for an essential role of tetraspanin in ROS generation.

## Author Summary

ROS are highly reactive molecules, which can be inappropriately produced during aerobic metabolism or by exogenous stresses such as exposure to UV light and radiation. ROS interact with cellular components including nucleic acids, lipids, and proteins and irreversibly inhibit their functions. However, ROS are essential for innate host defense and multiple physiological processes and are generated by conserved NADPH oxidase (NOX) family enzymes. The release of ROS by ROS generator enzymes must be properly controlled, as chronic oxidative stress can cause an imbalance of the redox state and is often associated with disease and aging. Using *C. elegans* as a model, we identified a tetraspanin (TSP-15) protein as a new key component of the ROS generation system controlled by dual oxidase (BLI-3), a unique NOX isozyme in *C. elegans*. Mutants of both *bli-3* and *tsp-15* developed the same defects in extracellular matrix cross-linking. Using a combination of genetics and reconstitution experiments in mammalian cells, we have demonstrated a novel requirement of tetraspanin for dual oxidase-dependent ROS generation via complex formation at the cell surface.

## Introduction

Reactive oxygen species (ROS) are considered deleterious by-products of aerobic metabolism that inflict oxidative damage in organisms, and have been associated with numerous diseases and aging. ROS are produced in phagocytic and non-phagocytic cells and function to eliminate invading microbes [1], [2]. The physiological generation of ROS is directed by the NADPH oxidase (NOX) family of enzymes, which are highly conserved integral membrane proteins comprising seven members in mammals (NOX1–NOX5, DUOX1, and DUOX2) [3]–[5]. Studies of the NOX family have uncovered multiple biological functions of ROS in developmental processes, apoptosis, protein modification, cellular signaling and are well documented in host defense mechanisms [1], [6]–[8]. Dual oxidases (DUOX) were originally identified as thyroid oxidases, key H_2_O_2_ generators for the iodination of tyrosine in thyroid hormone precursors during thyroid hormone biosynthesis [9]–[11]. Whereas most NOX enzymes release superoxide, DUOXs release only H2O2 at the cell surface in physiological conditions, by rapid dismutation of intermediate superoxide [12], [13]. Mutations in the *DUOX2* gene are linked to congenital hypothyroidism in humans and mice [14], [15]. DUOX-mediated H_2_O_2_ production is also crucial for other biological processes, such as extracellular matrix formation [16]–[18], innate immunity [19]–[22], and wound healing [23], [24]. In *C. elegans*, BLI-3 encodes a nematode orthologue of DUOXs that is essential for exoskeletal development via tyrosine cross-linking [17], [25]–[27], but which also functions in pathogen-induced ROS production [28]–[30].

The activity of the catalytic core of NOX enzymes is post-translationally controlled by the recruitment of regulatory subunits to the plasma membrane [5], [31], [32]. In contrast to NOX isozymes, the current understanding of the regulation of DUOX proteins is unclear, despite the identification of maturation factors (DUOXA) [33]. Dual oxidase maturation factors (DUOXA1 and DUOXA2) heterodimerize with DUOX and contribute to its intracellular trafficking [34]–[36]. In the absence of DUOXA, DUOX is not recruited to the plasma membrane and is inactive [37], [38]. DUOX1 preferentially dimerizes with DUOXA1, while DUOX2 preferentially forms dimers with DUOXA2 to achieve maximum activity [35], [36]. Similar to *DUOX2*, missense mutations in the *DUOXA2* gene were found in patients with congenital hypothyroidism [39]. In addition to regulation by DUOXA, DUOX contains EF-hand motifs in the cytoplasmic region, and calcium (Ca^2+^) stimulation is essential for H_2_O_2_ production.

Tetraspanins are integral membrane proteins defined by conserved secondary structures including four transmembrane regions, short cytoplasmic tails at the N- and C-termini, and small and large extracellular loops containing conserved cysteine residues [40], [41]. They constitute a widely expressed protein superfamily with 33 members in humans. Tetraspanin acts as a molecular facilitator by association and orchestrates a number of other proteins and tetraspanins in specialized membrane microdomains, termed tetraspanin-enriched microdomains (TEMs). TEMs are a distinct class of membrane microdomains with their own biochemical characteristics. TEMs are reportedly a new type of signaling platform involved in cell-cell communication [42]–[44]. Numerous studies have shown the functional relevance of tetraspanins in cell adhesion, motility, membrane fusion and antigen presentation. Additionally, tetraspanins are implicated in pathological processes such as tumor malignancy and infectious diseases [45], [46]. In addition to modification of integrin-mediated cellular functions [47], tetraspanins are important for the proteolytic regulation of β-amyloid precursor protein (β-APP) and Notch, and the specificity of Norrin/β-catenin signaling by regulating its receptor, Frizzled-4 [48]–[50].

Evidence from model organisms and inherited human diseases has provided insight into tetraspanin functions *in vivo*. Previously we demonstrated that a reduction in tetraspanin *tsp-15* function, led to exoskeletal deficiencies and lesions in the maintenance of barrier function [51]. The exoskeleton (cuticle) of *C. elegans* is mainly composed of collagen, synthesized and secreted from the apical surface of underlying epidermal cells (hypodermis) [52]. In the current study, we have identified a series of mutations in genes that are components of the nematode DUOX system. Based on our evidence, we propose that tetraspanin is a newly identified regulatory component of the DUOX system for H_2_O_2_ production.

## Results

### Identification of DUOX system mutants resembling the *tsp-15* mutant

**Figure 1:**
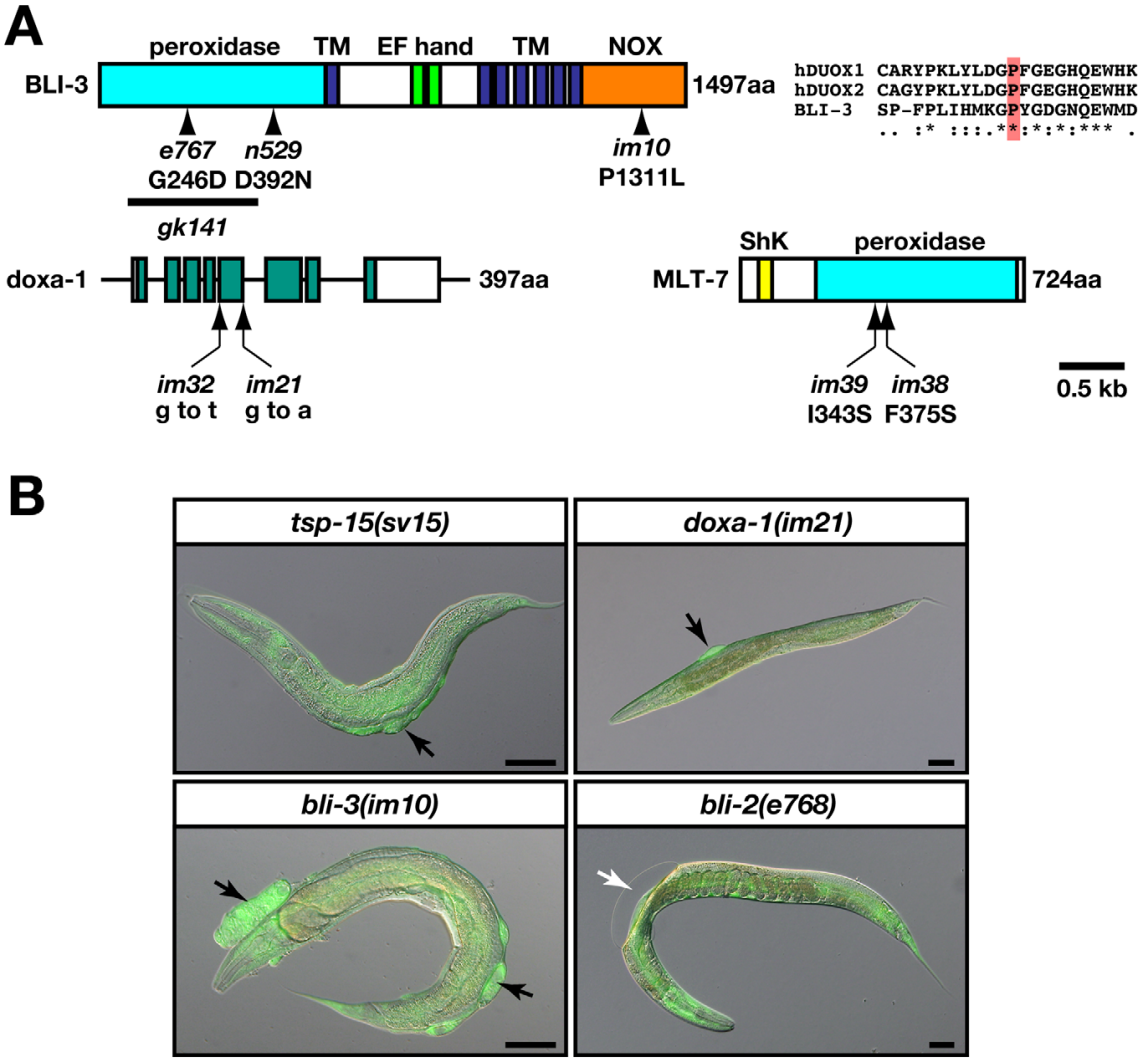
*bli-3* and *doxa-1* mutants are similar to the *tsp-15* mutant. (A) The structure of the gene/proteins related to *tsp-15* function. Schematic representation of the BLI-3 and MLT-7 protein with functional domain, and the genomic structure of the *doxa-1* gene. The *im10*, *im21*, *im32*, *im38* and *im39* mutations are indicated. Previously identified missense mutations in the *bli-3* gene including *e767* (glycine to aspartic acid at 246) and *n529* (aspartic acid to asparagine at 392) are shown. The bold line indicates the region of the *gk141* deletion allele. The *im10* mutation has a leucine instead of a proline at position 1311 within the NOX domain. TM and NOX refer to the transmembrane and NOX domains, respectively. The *im21* mutation is characterized by a G to A transition in the splice donor site at the fifth intron. The *im32* mutation is a G to T transversion in the splice acceptor site at the fourth intron. The *im38* and *im39* alleles are indicated in the MLT-7 protein. Both alleles contain the missense mutations in the peroxidase domain. (B) *bli-3(im10)* and *doxa-1(im21)*, but not *bli-2(e768)* are similar to *tsp-15(sv15)*. Hypodermal expression of GFP driven by *dpy-7p::gfp* in the mutants revealed an unusual accumulation of cellular materials in the blisters of *bli-3*, *doxa-1* and *tsp-15* mutants (indicated by black arrows), but not in *bli-2* mutants (indicated by the white arrow). The scale bars represent 50 µm.

The splicing error mutation, *sv15*, within *tsp-15* causes a reduction in function of *tsp-15* [51]. We characterized other *tsp-15* mutants and found that those with deletions within *tsp-15* coding regions were lethal to embryos (Table 1, Figure S1, Table S1, Video S1). We screened for novel mutants similar to the *tsp-15* hypomorph mutant to obtain clues for the *tsp-15* mutant phenotype. We have shown that *tsp-15(sv15)* mutants have a distinct blister phenotype compared with classical *bli* mutants, that were classified by *dpy-7p::gfp* expression (Figure 1B) [51]. Both N2 and OB43 *imIs1[dpy-7p::gfp]* strains were mutated and screened to exclude typical *bli* mutants [53]. We isolated thirteen alleles classified in four independent complementation groups (CG1–CG4; Table S2). Through single nucleotide polymorphism (SNP) mapping, DNA sequencing, RNAi, and complementation assays, we identified five mutations (Table S2). All identified responsible genes encoded DUOX or related proteins (Figure 1). The *im10* mutation in CG1 is a missense mutation in the F56C11.1 gene encoding *bli-3/CeDuox-1*, a homologue of mammalian dual oxidases (Figure 1A) [17]. A conserved proline at position 1311 in the NOX domain was changed to leucine (P1311L) in *im10* mutants. The *gk141* was thought to have a deletion in the *bli-3* region, and hT2-balanced heterozygotes produced *gk141/hT2* adults, indicating that *gk141/gk141* homozygotes were embryonically lethal (Table 1, Figure S2A). The *im21* and *im32* mutations in CG3 were located in the splicing site of the C06E1.3 gene (Figure 1A), possibly causing premature termination. Amino acid comparisons implied that C06E1.3 is a nematode homologue of DUOXA and essential for maturation and membrane targeting of DUOX (Figure S3) [33]; we named this gene *doxa-1*. Both *im38* and *im39* in CG4 were identified as missense mutations in ZK430.8, reported as *mlt-7* (Figure 1A, Figure S2C) [25]. Mutations *im38* and *im39* caused a change in the conserved isoleucine at 343 to serine, and phenylalanine at 375 to serine in the peroxidase domain. MLT-7 is a heme peroxidase and essential for cuticle biogenesis in combination with BLI-3 [25].

**Table 1:** The *tsp-15*, *doxa-1*, and *bli-3* genes are in the same genetic pathway.

*tsp-15* hypomorph mutant and rescue analysis	
Genotype	WT-like (%)*
*tsp-15(sv15)*	1.2
*tsp-15(sv15); imEx13[dpy-7p::HisXp::tsp-15]*	81.0
*tsp-15(sv15); imEx14[tsp-15::gfp]*	70.0
*tsp-15(sv15); imEx144[bli-3]*	1.10
*tsp-15(sv15); imEx113[doxa-1::venus]*	0
*tsp-15(sv15); imEx147[bli-3, doxa-1::venus]*	68.9

*tsp-15* deletion mutant and rescue analysis	
Genotype	Survived (%)*
*tsp-15(gk201)*	100 (WT)
*tsp-15(ok854)*	0 (Emb)
*tsp-15(ok881)*	0 (Emb)
*tsp-15(tm1666)*	0 (Emb)
*tsp-15(ok854); imEx64[dpy-7p::HisXp::tsp-15]*	93.6
*tsp-15(ok881); imEx65[dpy-7p::HisXp::tsp-15]*	94.3
*tsp-15(tm1666); imEx67[dpy-7p::HisXp::tsp-15]*	90.4
*tsp-15(ok881); imEx147[bli-3, doxa-1::venus]*	1.46 (Bli/Dpy)

*bli-3* mutant and rescue analysis	
Genotype	WT-like (%)*
*bli-3(e767)*	0
*bli-3(gk141)*	0
*bli-3(im10)*	0
*bli-3(n529)*	4.9
*bli-3(e767/im10)*	0
*bli-3(e767/gk141)*	0
*bli-3(gk141); imEx115[dpy-7p::bli-3]*	62.9
*bli-3(e767); imEx144[bli-3]*	95.7
*bli-3(im10); imEx144[bli-3]*	95.8
*bli-3(gk141); imEx144[bli-3]*	92.9
*bli-3(e767); imEx13[dpy-7p::HisXp::tsp-15]*	0.72
*bli-3(n529); imEx13[dpy-7p::HisXp::tsp-15]*	10.3
*bli-3(e767); imEx113[doxa-1::venus]*	0
*bli-3(e767); imEx148[dpy-7p::HisXp::tsp-15, doxa-1::venus]*	0

*doxa-1* mutant and rescue analysis	
Genotype	WT-like (%)*
*doxa-1(im21)*	0 (Emb/Bli/Dpy)
*doxa-1(im21); imEx114[dpy-7p::doxa-1]*	90.0
*doxa-1(im21); imEx113[doxa-1::venus]*	97.6
*doxa-1(im21); imEx149[bli-3, dpy-7p::HisXp::tsp-15]*	0

WT; wild-type, Emb; embryonic lethal, Bli; blister, Dpy; dumpy.

* >100 animals were counted for each strain.

The *bli-3*, *doxa-1*, and *mlt-7* mutants were rescued by their own cDNA driven by a hypodermis-specific *dpy-7* promoter (Table 1, Figure S2). For *im21*, Venus-tagged *doxa-1* at the C-termini (*doxa-1::venus*, Figure S4A) driven by the *doxa-1* promoter effectively rescued the phenotype (Table 1, Figure S2B, Figure S4B). The *doxa-1::venus* transgene was expressed in the hypodermis, and other tissues such as the pharynx, uterus, gonad, and vulva (Figure S4B).

### Involvement of *tsp-15* in *bli-3* pathway

**Figure 2:**
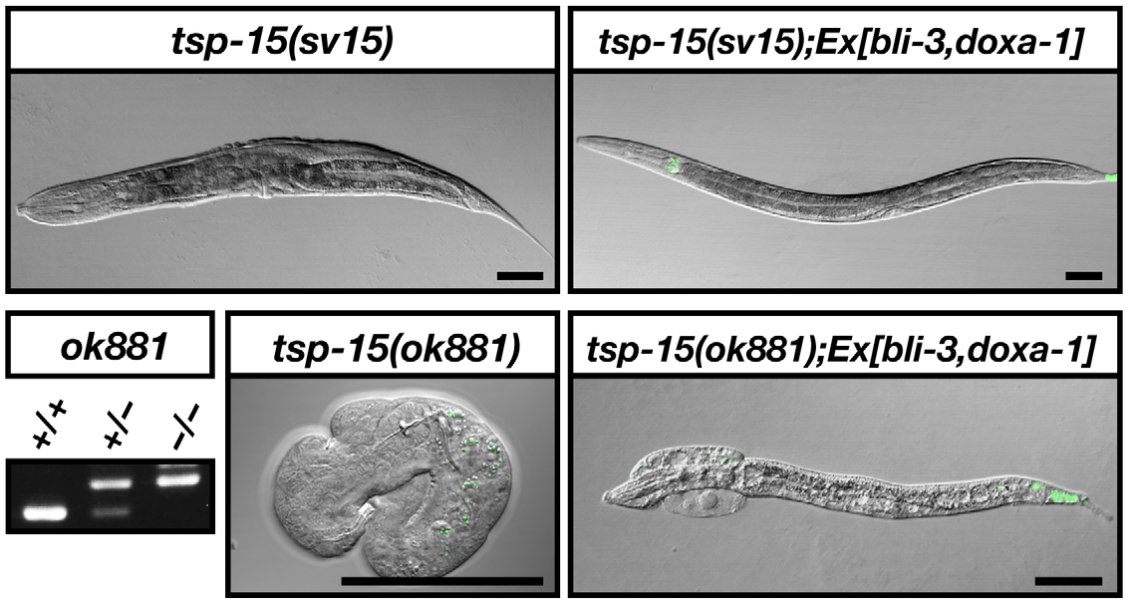
TSP-15 function is compensated with BLI-3 system. Restoration of the *tsp-15* defect by co-expression of *bli-3* and *doxa-1*. Co-expression of *bli-3* and *doxa-1* in the *tsp-15(sv15)* hypomorph mutant effectively rescued the phenotype of *sv15*. The embryonically lethal *tsp-15(ok881)* null mutant was partially rescued and developed into larvae *via bli-3* and *doxa-1* co-expression. Green fluorescence at the tail tip represents expression of the *lin-44::gfp* injection marker. The *ok881* deletion homozygosity of surviving transgenic larvae was confirmed by genomic PCR. The scale bars represent 50 µm.

To investigate the genetic relationship between *tsp-15* and newly isolated mutants, we performed mutant rescue assays (Figure 2, Table 1). Over-expression of *tsp-15* did not restore the defects of the *bli-3* and *doxa-1* mutants. Over-expression of *bli-3* as well as *doxa-1* alone did not rescue the *tsp-15* mutant. Co-expression of *bli-3* and *doxa-1* in the *tsp-15* hypomorph mutant effectively rescued the cuticle deficiency (Figure 2, Table 1). In contrast, *bli-3* and *doxa-1* co-expression in the *tsp-15* null mutant resulted in partial rescue of the lethal phenotype. In approximately 1.5% of transgenic animals, embryonic lethality was recovered and the larvae showed a *tsp-15* hypomorph mutant-like morphology (Figure 2), which has not been observed in *tsp-15* null mutants previously.

### Decreased dityrosine levels in *tsp-15* mutants

**Figure 3:**
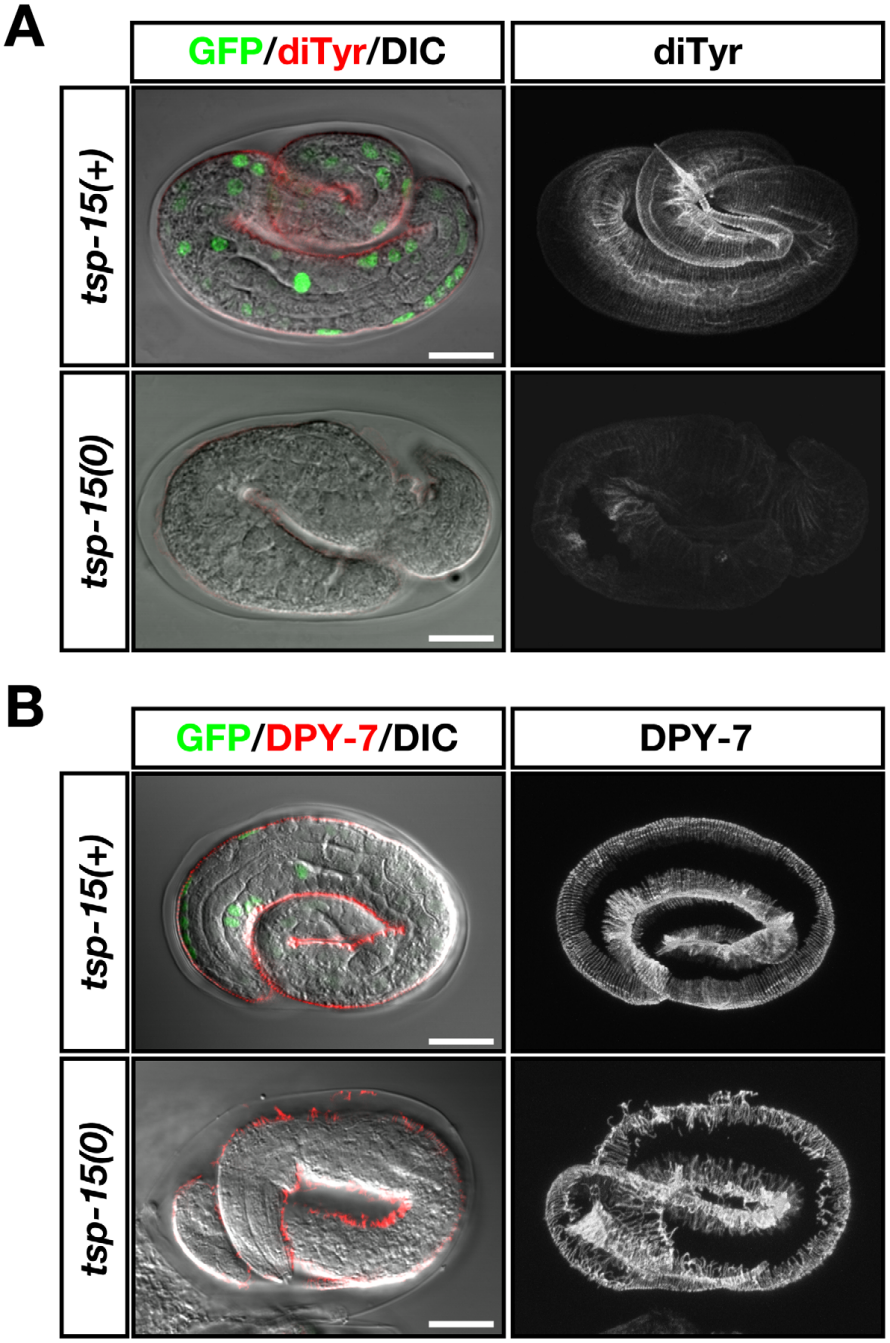
Deterioration of dityrosine in the *tsp-15* mutant. (A) Representative immunofluorescent images showing the distribution of dityrosine in embryos of the *tsp-15(ok854)* null mutant. Embryos were obtained from the OB129 strain, which was the *tsp-15(ok854)* mutant rescued by a *tsp-15p::(His)^6^Xp::tsp-15* extrachromosomal array. Nuclear GFP fluorescence by *sur-5::gfp* defined the rescued (*tsp-15(+)*) or spontaneously array-lost (null; *tsp-15(0)*) embryo. Micrographs on the left show merged Nomarski images exhibiting GFP and dityrosine immunolocalization. Right panels show the reconstruction of confocal images for dityrosine distribution in the same embryo that is displayed on the left. In *tsp-15(+)* normal embryos, dityrosine localization showed a regular pattern representing the cuticle surface structure. Fluorescence intensity was severely deteriorated in *tsp-15(0)* embryos. Scale bars indicate 10 µm. (B) DPY-7 localization was compared under the same conditions as in (A). In *tsp-15(+)* embryos, DPY-7 localized as regular bands in the cuticle. In the *tsp-15(0)* embryo, the expression of DPY-7 was comparable to the normal embryo despite its disorganized pattern. Scale bars indicate 10 µm.

BLI-3 is reported as a key enzyme for the generation of H_2_O_2_ for tyrosine cross-linking in the cuticle since the level of di- and tri-tyrosine formation is reduced in the cuticle of *bli-3(RNAi)* animals [17]. To examine distribution of cross-linked tyrosine in the exoskeleton of *tsp-15* mutants, we carried out immunohistochemical analysis using anti-di-tyrosine antibody. We also checked the endogenous distribution of DPY-7 (a nematode collagen) in *tsp-15* mutants by immunostaining. In the normal embryo, di-tyrosine was distributed over the entire cuticle representing the body surface structure, whereas di-tyrosine formation was severely reduced in *tsp-15* null embryos (Figure 3A). In contrast, the level of collagen found in *tsp-15* null embryos, examined *via* DPY-7, was comparable to *tsp-15(+)* embryos, although distribution was severely disturbed (Figure 3B). Thus, cuticle collagen is likely synthesized and secreted from the hypodermis correctly, but a failure of cross-linking of secreted collagen results in fragility of the cuticle in *tsp-15* mutants.

### BLI-3 activity depends on both TSP-15 and DOXA-1

**Figure 4:**
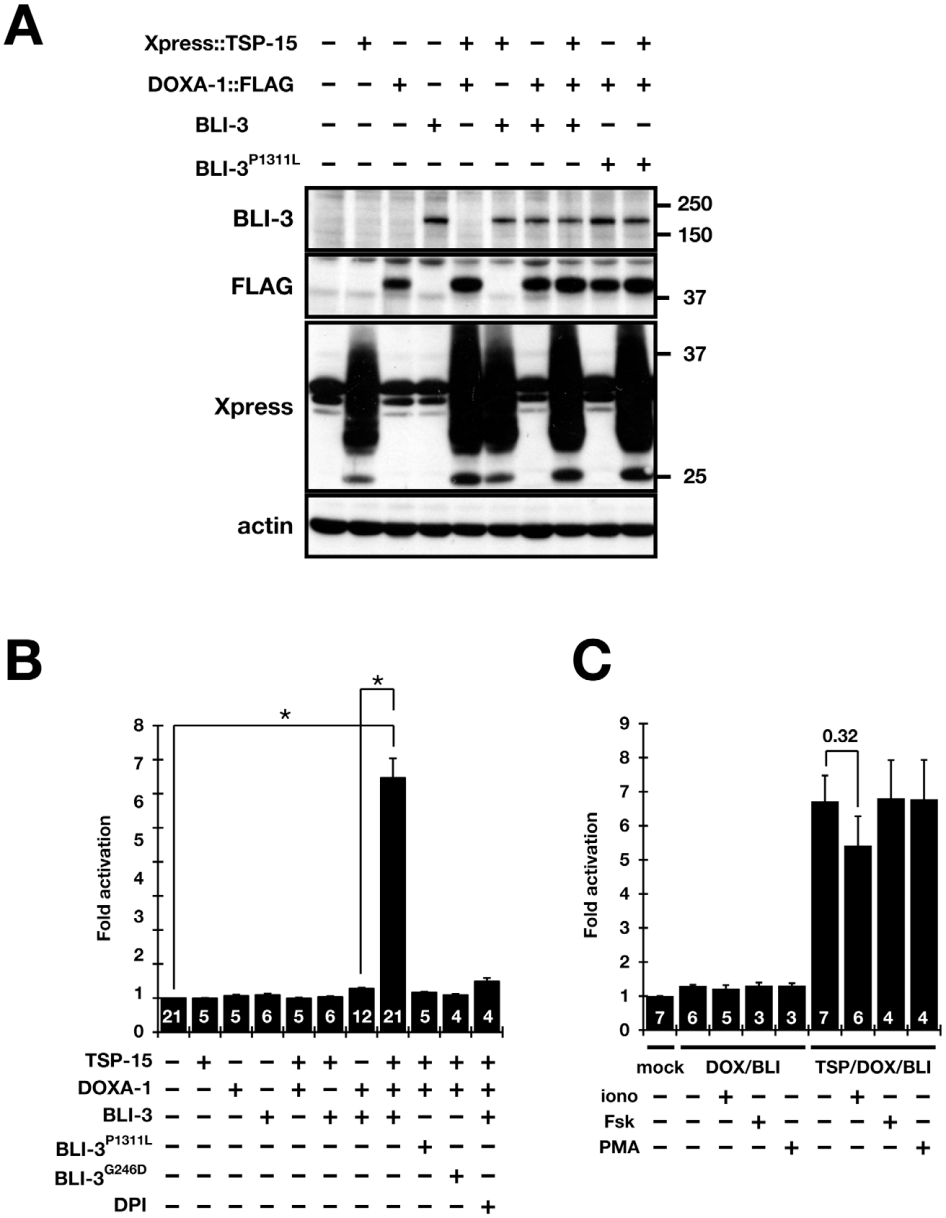
Both TSP-15 and DOXA-1 are required for H_2_O_2_ production by BLI-3 in mammalian cells. (A) Immunoblot analysis of the expression of Xpress-tagged TSP-15, FLAG-tagged DOXA-1, BLI-3 and BLI-3^P1311L^ in HT1080 stable transfectants. Xpress-tagged TSP-15 (30 kDa) is highly glycosylated (Figure S5). (B) Extracellular H_2_O_2_ production from stable transfectants. Fold-activation compared with non-transfected HT1080 cells was determined. Only cells expressing TSP-15, DOXA-1, and BLI-3 (HT1080^TDB^) significantly generated H_2_O_2_. A 10 µM concentration of DPI inhibited H_2_O_2_ production in HT1080^TDB^ cells. BLI-3 carrying the G246D or P1311L mutation did not release H_2_O_2_. The graph shows the means ± SEM. The numbers in the bars indicate the number of independent experiments (*n*). *P<10^−9^. (C) BLI-3 was not activated by mammalian DUOX stimulators. Ionomycin (iono, 1 µM), forskolin (Fsk, 1 µM), or phorbol 12-myristate 13-acetate (PMA, 1 µM) was added to HT1080^DB^ and HT1080^TDB^ cells during culture. The graph shows the means ± SEM. The numbers in the bars indicate the number of independent experiments (*n*).

DUOX was originally identified as a hydrogen peroxide generator for thyroid hormone synthesis [9], [11] and DUOXA is essential for DUOX targeting to the plasma membrane [33]. We produced stable transfectants of TSP-15, BLI-3 and DOXA-1 in human HT1080 cells (Figure 4A) to confirm the roles of TSP-15 and DOXA-1 for BLI-3. Release of H_2_O_2_ into the culture medium was measured in the absence of other *C. elegans* proteins. Extracellular H_2_O_2_ from HT1080^B^ and HT1080^TB^ cells was almost equivalent to basal activity (see Materials and Methods for description of stable transfectants). Unlike the mammalian DUOX system, BLI-3 was only slightly activated by co-expression of DOXA-1 in HT1080^DB^ cells. In addition to DOXA-1, concomitant expression of TSP-15 strongly enhanced production of H_2_O_2_ in HT1080^TDB^ cells (Figure 4B). The generation of H_2_O_2_ was blocked by the flavoprotein inhibitor diphenyleneiodonium (DPI), indicating that DUOX was involved in enhanced H_2_O_2_ production in TSP-15-transduced cells. We concluded that BLI-3 requires TSP-15 and DOXA-1 for proper function. BLI-3^P1311L^ and BLI-3^G246D^ identical to the *im10* or *e767* mutation, respectively, resulted in decreased H_2_O_2_ production (Figure 4B). The same results were observed in other independently established stable cell lines, and by transient expression in COS-7 and HeLa cells (data not shown). Regulation of Ca^2+^ characteristically elicits DUOX activity as a thyroid hormone synthesizer. BLI-3 did not require calcium stimulation to produce H_2_O_2_ in HT1080^TDB^ cells, and HT1080^DB^ cells were not activated by calcium stimulation either (Figure 4C). This may be due to the fact that the critical amino acid residues for Ca^2+^-binding are poorly conserved in the EF-hand motifs of BLI-3 proteins [17]. Furthermore, BLI-3 was not activated by forskolin (fsk) and phorbol 12-myristate 13-acetate (PMA), which have previously been reported to be mammalian DUOX stimulators (Figure 4C) [54].

### TSP-15 and DOXA-1 associate with BLI-3

**Figure 5:**
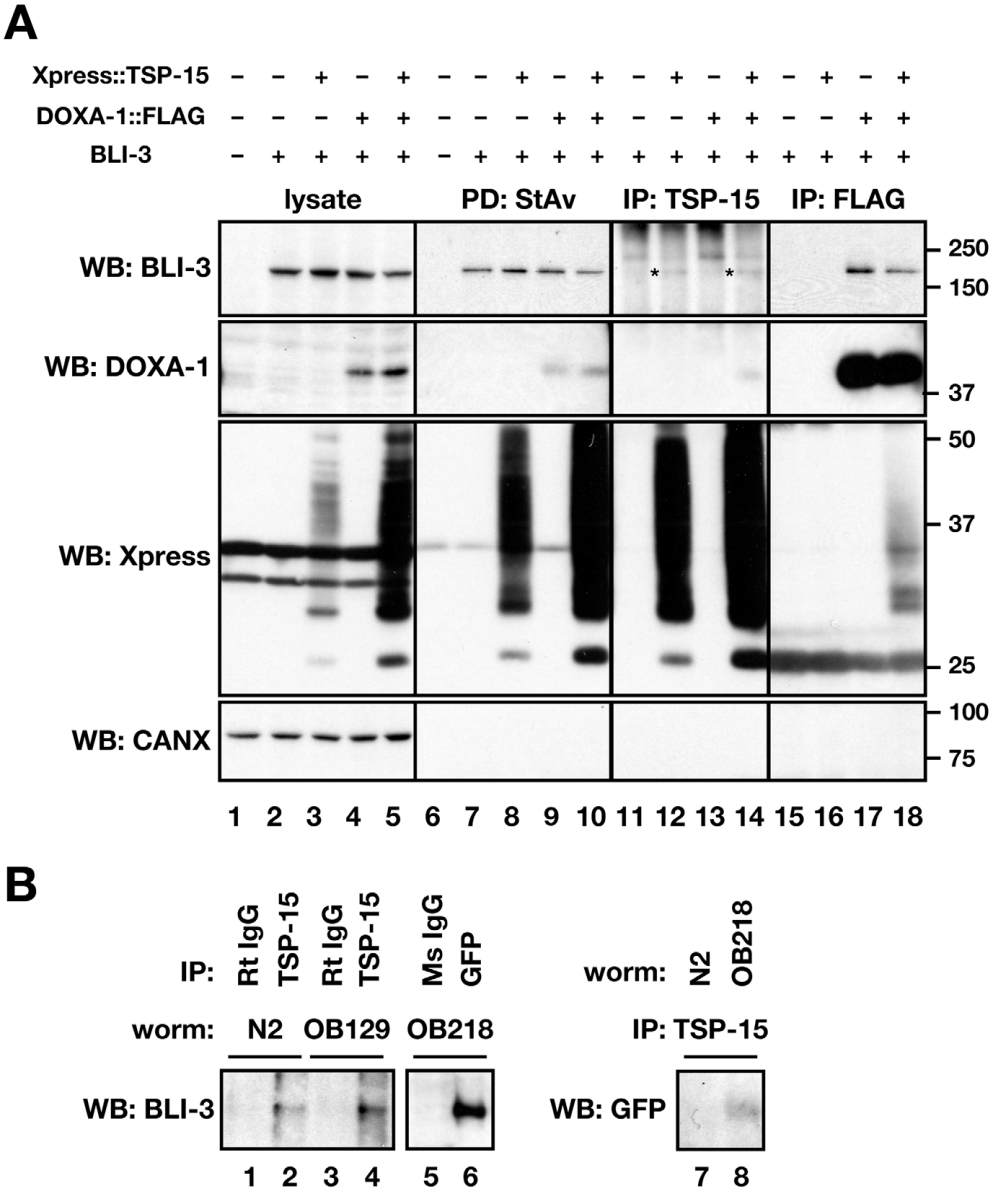
Direct association of BLI-3 with TSP-15 and DOXA-1. (A) Direct association of BLI-3 with TSP-15, and BLI-3 with DOXA-1. BLI-3 was transiently expressed in COS-7 stable transfectants expressing Xpress::TSP-15, DOXA-1::FLAG, or both. Cell surface proteins were labeled with biotin. A 1% CHAPS cell lysate was used for immunoprecipitation or pull-down assay with anti-TSP-15 antibody, anti-FLAG antibody or streptavidin. BLI-3 was co-immunoprecipitated with both TSP-15 (indicated by asterisks) and DOXA-1, and TSP-15 and DOXA-1 were co-immunoprecipitated. Bands above asterisks are non-specific. Cell surface localization of BLI-3 was independent of TSP-15 and DOXA-1. ER-resident calnexin (CANX) was not detected on the cell surface. BLI-3 expression was not affected by TSP-15 and DOXA-1. (B) Direct association of BLI-3 with TSP-15, BLI-3 with DOXA-1, and TSP-15 with DOXA-1 was confirmed in *C. elegans*. *Xpress::tsp-15* was expressed in OB129, and *doxa-1::venus* is expressed in the OB218 transgenic strain. Venus-tagged DOXA-1 was analyzed with anti-GFP antibody. BLI-3 was co-immunoprecipitated with endogenous and Xpress-tagged TSP-15 and Venus-tagged DOXA-1 in 1% Triton-X100 cell lysates. Endogenous TSP-15 also associated with DOXA-1::Venus. Normal rat and mouse IgG was used as a negative control.

Tetraspanins form protein complexes with a number of other molecules. We performed co-immunoprecipitation assays to determine whether TSP-15 associates with BLI-3 and DOXA-1. BLI-3 was transiently expressed in COS-7 cells where TSP-15 and/or DOXA-1 was stably expressed. As a result, BLI-3 co-immunoprecipitated with DOXA-1 (Figure 5A; lanes 17 and 18) and TSP-15 (Figure 5A, lanes 12 and 14). Co-immunoprecipitation of BLI-3 and DOXA-1 was independent of TSP-15 expression (Figure 5A; lane 17). TSP-15 co-immunoprecipitated with BLI-3 in the absence of DOXA-1 (Figure 5A; lane 12). In addition, TSP-15 and DOXA-1 association was also observed (Figure 5A; lane 14 and 18, Figure S6), indicating that TSP-15, DOXA-1, and BLI-3 form protein complexes. TSP-15 was not co-immunoprecipitated with over-expressed EGF receptor under the same conditions (data not shown). We also verified the same molecular interaction in transgenic animals. In *doxa-1::venus* transgenic worms (Figure S4B), BLI-3 co-immunoprecipitated with the DOXA-1::Venus fusion protein (Figure 5B; lane 6). Endogenous and tagged TSP-15 associated with BLI-3 (Figure 5B; lanes 2 and 4) and with DOXA-1::Venus (Figure 5B; lane 8). We concluded that TSP-15, DOXA-1 and BLI-3 form a complex.

BLI-3 expression levels in total cell lysates were unaffected by DOXA-1 and TSP-15 expression (Figure 5A; lanes 2–5). We examined surface expression of BLI-3 using biotin labeling. The amount of BLI-3 protein at the cell surface was not augmented by TSP-15 (Figure 5A; lanes 6–10). BLI-3 was also recruited to the plasma membrane without TSP-15. Therefore, TSP-15 is dispensable for BLI-3 targeting. It is reported that membrane targeting of mammalian DUOX depends on DUOXA in a system using non-thyroidal cells [33]–[36]; however, BLI-3 was recruited to the plasma membrane without DOXA-1 in our system.

### BLI-3 activation by TSP-15 at the cell surface

**Figure 6:**
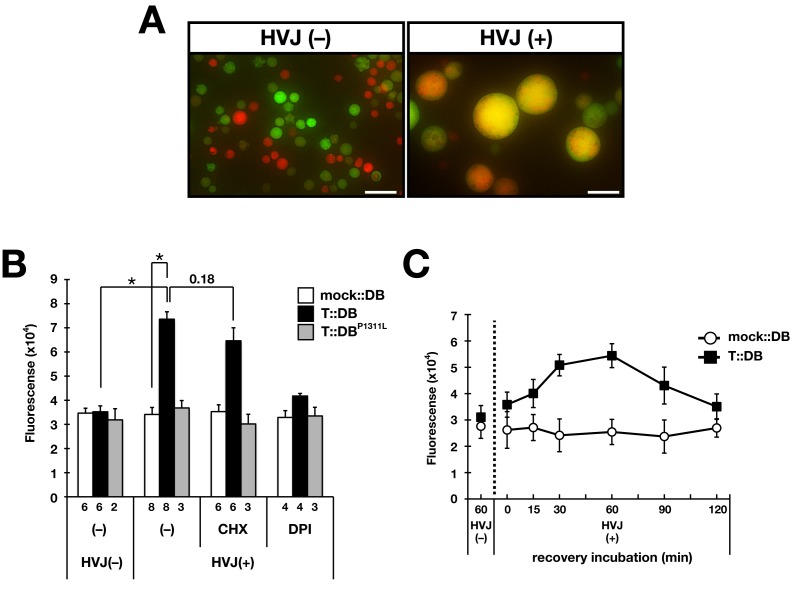
Requirement of TSP-15 for reconstitution of BLI-3 function at cell surface. (A) HVJ-mediated cell fusion. GFP-expressing HT1080 cells and HT1080^DB^ cells labeled with Cell Tracker Orange were fused with HVJ. Under HVJ(+) conditions, fused cells were large compared with HVJ(−) cells and exhibited yellow/orange fluorescence. Scale bar indicates 50 µm. (B) HT1080^DB^ cells fused with HT1080^T^ cells (T::DB) produced H_2_O_2_, which was inhibited by DPI. Mock::DB and T::DB^P1311L^ fusion cells did not produce H_2_O_2_. Treatment of T::DB fusion cells with 10 µg/ml cycloheximide (CHX) did not inhibit H_2_O_2_ production. The graph shows the means ± SEM. The number of independent experiments is indicated. *P<10^−6^. (C) Rapid H_2_O_2_ production from T::DB fusion cells. The recovery time after the fusion event was examined to determine when fusion cells acquired the ability to produce H_2_O_2_. Maximum H_2_O_2_ production was observed at 30–60 min, although production was observed at 15 min post-fusion. The graph shows the means ± SEM (*n* = 3).

The molecular requirement of TSP-15 in the BLI-3 system is possibly due to formation of a complex at the plasma membrane, therefore BLI-3 could be activated by TSP-15 localized at the cell surface. We assessed this hypothesis through cell fusion-based analysis (Figure 6A). TSP-15-expressing cells (HT1080^T^) and DOXA-1/BLI-3-expressing cells (HT1080^DB^), both of which did not produce H_2_O_2_ (Figure 4B), were fused utilizing Sendai virus (HVJ). After the cell fusion reaction, extracellular H_2_O_2_ production from fused cells was measured. The fused cells (T::DB) produced H_2_O_2_, and the production was inhibited by DPI treatment (Figure 6B). In contrast, BLI-3 carrying the P1311L mutation did not result in H_2_O_2_ production. Inhibition of *de novo* protein synthesis by cycloheximide (CHX) resulted in a slight decrease in H_2_O_2_ producing activity in T::DB fusion cells (Figure 6B). These results indicate that TSP-15 did not promote BLI-3 protein expression, but existing TSP-15 at the cell surface was sufficient to activate BLI-3 for H_2_O_2_ production. The capability of H_2_O_2_ production was rapidly acquired after cell fusion. H_2_O_2_ was immediately detectable in T::DB fusion cells 15 min after the fusion reaction (Figure 6C), indicating that alternatively derived TSP-15, BLI-3 and DOXA-1 rapidly formed functional units at the cell surface.

## Discussion

**Figure 7:**
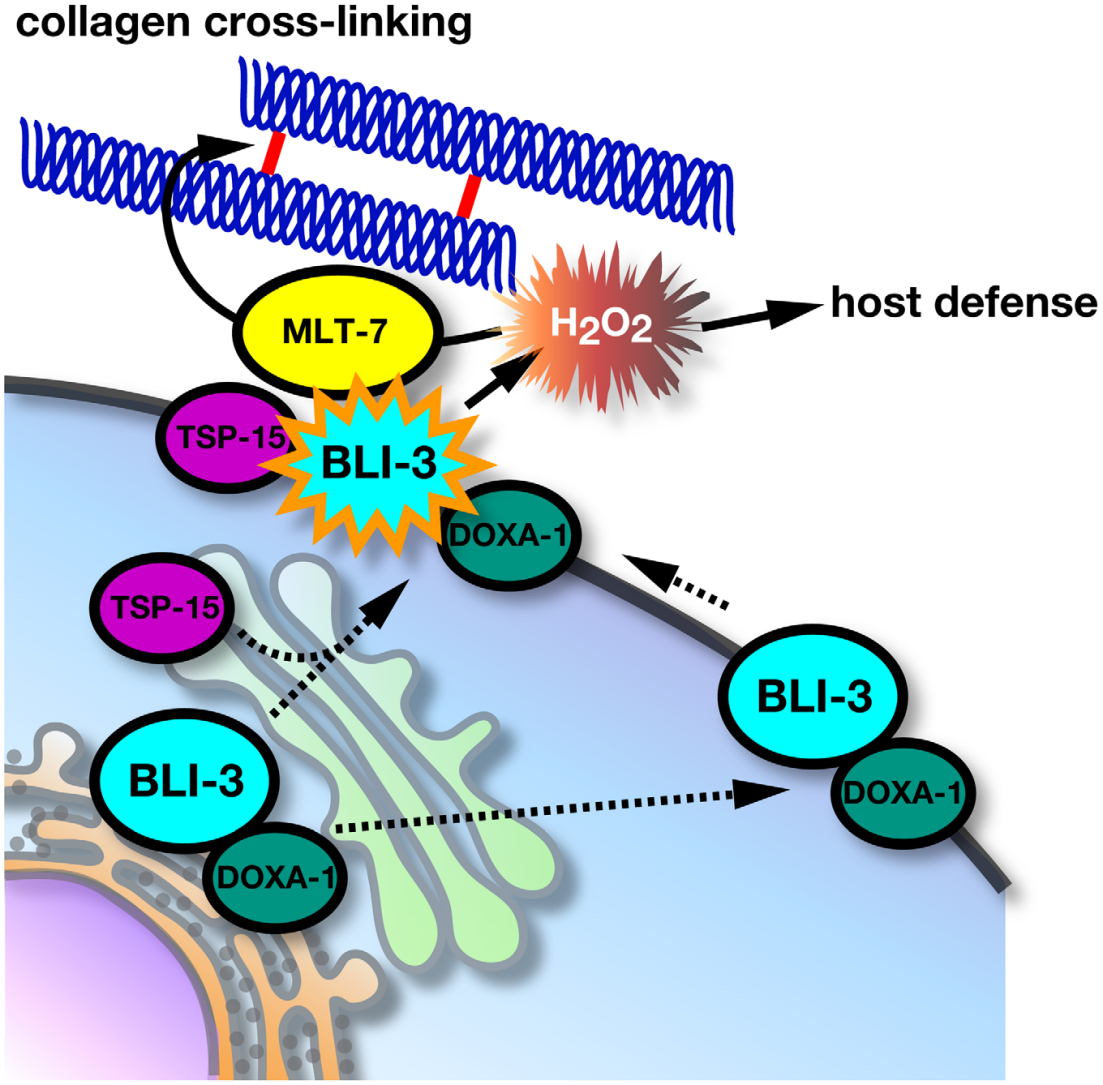
Molecular regulation of BLI-3 by TSP-15 and DOXA-1. TSP-15 and DOXA-1 are essential for H_2_O_2_ production by BLI-3. TSP-15 associates with BLI-3 at the cell surface or during trafficking. The role of DOXA-1 in BLI-3 targeting to the plasma membrane remains elusive. BLI-3/DOXA-1 complexes at the cell surface are inactive, but recruiting to the tetraspanin-microdomain facilitates the formation of a functional unit for generation of H_2_O_2_ that is utilized by innate host immunity, and cross-linking of extracellular matrix with peroxidase (MLT-7).

Organisms have developed regulatory systems to control ROS generation in host defense and cellular signaling. For mammalian DUOX proteins, association with a maturation factor (DUOXA) for targeting to the plasma membrane, Ca^2+^ regulation *via* EF-hand motifs, and PKA- or PKC-mediated phosphorylation were identified as regulatory systems. We propose that the tetraspanin protein is a novel component of the DUOX system for ROS generation (Figure 7). Using *C. elegans* as a model organism, we identified a series of genes for ROS generation in which mutants exhibited a phenotype resembling the tetraspanin *tsp-15* mutant. The genes *bli-3*, *doxa-1*, and *mlt-7* were, respectively, the homologues of mammalian DUOX, DUOXA and peroxidase, and mutants of these displayed the same cuticle deficiency (Figure 1, Figure 2, Figure S1, Figure S2). The reason for this cuticle disorganization is deterioration of tyrosine cross-linking in cuticle development as shown in *bli-3* knockdown-animals (Figure 3) [17]. We showed that the *tsp-15* mutant was rescued by simultaneous over-expression of *bli-3* and *doxa-1*, implying that these three genes are part of the same genetic pathway (Figure 1, Figure 2). Reconstitution of BLI-3, TSP-15 and DOXA-1 in mammalian cells demonstrated that H_2_O_2_ generation by BLI-3 was dependent on TSP-15 as well as DOXA-1 (Figure 4).

It was hypothesized that TSP-15 might enhance BLI-3 protein levels by elevating protein expression or promoting targeting to the cell surface; however, BLI-3 and DOXA-1 protein expression levels were comparable, with or without TSP-15 expression. TSP-15 expression did not affect BLI-3 expression at the cell surface, and did not enhance the association of BLI-3 and DOXA-1. This implies that the role of TSP-15 in the BLI-3 system is not just augmentation of its expression. Our observations support this notion, since over-expression of *bli-3* and *doxa-1* resulted in incomplete rescue of *tsp-15* null mutants. In the *sv15* hypomorph mutant, *tsp-15* expression was reduced, and expressed at 10% of wild-type levels (data not shown), therefore the BLI-3 system recovered to produce adequate H_2_O_2_ by concomitant over-expression of *bli-3* and *doxa-1*. If the up-regulation of BLI-3 activity by TSP-15 is quantitative, *tsp-15* null mutants should be completely rescued by BLI-3/DOXA-1, however this was not the case. The molecular role of tetraspanin in the DUOX system is likely qualitative. We believe that TSP-15 up-regulates the activity of BLI-3 at the plasma membrane. Cell fusion-based analysis strongly supports this idea, since cells that acquired TSP-15 from other cells rapidly produced H_2_O_2_ even when protein synthesis was inhibited. During the HVJ-mediated fusion process, intracellular organelles were morphologically altered and repaired within 30 min [55]. We observed that H_2_O_2_ generation was initiated 15 min after recovery, suggesting that individually derived BLI-3/DOXA-1 and TSP-15 rapidly assembled at the cell surface, forming functional complexes. The lipid raft marker protein, flotillin, was rapidly assembled during cell fusion [56]. Inhibition of *de novo* protein synthesis did not affect H_2_O_2_ production in fusion cells, indicating that the existing TSP-15 at the cell surface is sufficient for facilitation of BLI-3 activity.

The molecular mechanisms of up-regulation are still unclear, but we showed that TSP-15, BLI-3 and DOXA-1 form complexes *in vitro* and *in vivo* (Figure 5). BLI-3 directly associates with DOXA-1, as demonstrated in mammalian DUOX and DUOXA. We also demonstrated the association between BLI-3 and TSP-15, and that this was independent of DOXA-1 expression. It is known that tetraspanin associates with a number of membrane proteins and forms large protein complexes at certain membrane microdomains. We speculate that TSP-15 may establish or maintain a specialized membrane microdomain that facilitates generation of H_2_O_2_ in conjunction with BLI-3. As reported for other NOX isozymes and their subunits, association with TSP-15 might induce a conformational change in BLI-3 to function properly. Alternatively, TSP-15 may support the recruitment of unknown factors at the membrane microdomain that are essential for BLI-3 activity. Although DOXA-1 is essential for H_2_O_2_ production by BLI-3, the role of DOXA-1 in the BLI-3 system is unclear. Unlike mammalian DUOX, BLI-3 was unexpectedly recruited to the plasma membrane in the absence of DOXA-1. DOXA-1 might not regulate BLI-3 trafficking in the DUOX system in *C. elegans*, but we cannot exclude the possibility that expression of *C. elegans* proteins in mammalian cells may cause dysregulation in BLI-3 trafficking. Further investigation is needed to clarify the molecular functions of DOXA-1 in the BLI-3 system. In addition, unlike the mammalian DUOX system, BLI-3 did not respond to various stimuli when reconstituted in mammalian cells (Figure 4C). Absence of negative regulatory factors may explain the constitutive active state of BLI-3 in the heterologous system. For instance, NOXA1 has an inhibitory role in stabilizing the inactive state of mammalian DUOX1 [57]. No NOXA1-like sequence has been found in *C. elegans*, but further investigation would clarify this hypothesis.

Reciprocal homology searches suggested that several human tetraspanins are related to TSP-15 with CD151 (TSPAN24) and TSPAN11 being the most closely related. However, we have not identified any mammalian tetraspanins that could be functionally substituted for TSP-15 in the *tsp-15* mutant [51], or for H_2_O_2_ production in the BLI-3/DOXA-1 reconstitution system (data not shown). It is also uncertain whether mammalian tetraspanins have a pivotal role in mammalian DUOX system. Mutations in tetraspanin genes have not been identified in patients suffering from congenital hypothyroidism. In contrast to other NOX isozymes, understanding the regulation of DUOX proteins is emerging. Our data clearly shows that tetraspanin is a new component for directing DUOX activity, contributing to greater understanding of the molecular mechanisms of ROS generation and disorders caused by impairment of ROS generation systems [58], [59].

## Materials and Methods

### Worm strains and culture

*C. elegans* was grown at 20°C on NGM plates as described previously [60]. The Bristol N2 strain was used as the wild type. Strains and their genotype used in this study are listed in Table S1.

### Mutant screening and identification

N2 or OB43 were mutated with 50 mM ethyl methanesulfonate or 5 mM ethyl nitrosourea for 4 h. F2 recessive mutants showing a *sv15* mutant-like phenotype were screened. SNPs between N2 and Hawaiian CB4856 strains were used for physical mapping of the alleles [61]. Mutations were determined by further DNA sequencing and confirmed by complementation tests, rescue assays by DNA transformation, and RNAi analyses (see Text S1). Mutants were outcrossed at least five times with N2.

### cDNA cloning and construction of vectors

Total RNA was isolated from mixed stages of N2 or mutant worms using TRIzol reagent (Invitrogen, Carlsbad, CA, USA). First strand cDNA was synthesized by ReverTra Ace (Toyobo, Japan). A 4.6 kb fragment of *bli-3*, 1.2 kb of *doxa-1*, and 2.2 kb of *mlt-7* full length cDNA was prepared by RT-PCR. The *bli-3^P1311L^* and *bli-3^G246D^* vectors were constructed by PCR-based site-directed mutagenesis. *doxa-1::venus* translational fusion construct (Figure S4A) contains a 3.1 kb genomic PCR fragment of the *doxa-1* 5′ flanking region and 2.2 kb *doxa-1* genomic coding region without a termination codon, which was cloned into the Venus translational fusion vector (a gift from Takeshi Ishihara, Kyusyu University). The (His)^6^Xpress-tagged *tsp-15* (*HisXp::tsp-15*), *bli-3*, FLAG-tagged *doxa-1* (*doxa-1::FLAG*) and *mlt-7* were sub-cloned under the control of the *dpy-7* promoter for hypodermis-specific expression in the mutant rescue assay [51] or into the pCx4 retroviral vector for transfection into mammalian cells [62]. A 34.2 kb fosmid clone, WRM065cD06, was purchased from Geneservice (Cambridge, UK). A 14.9 kb *Hae*II restriction fragment (nt 16368–31233) which contained the full *bli-3* coding region and also the 5′ flanking region was used for the rescue assay.

### Immunohistochemistry and microscopic imaging of embryos

Embryos were collected from gravid hermaphrodites and were immunostained as previously described [63]. Mouse anti-dityrosine (1C3; Nikken Seil Co., Ltd. Shizuoka, Japan), mouse anti-DPY-7 (a gift from Iain L. Johnstone, University of Glasgow) [64] were used at 1∶200 and 1∶500 dilutions, respectively. Alexa Fluor 488 or Alexa Fluor 546 (Invitrogen) conjugated anti-mouse IgG antibodies were used as secondary antibodies at 1∶500 dilutions. Confocal images were acquired with a LSM5 Pascal microscope (Zeiss, Germany). The three-dimensional projections were reconstructed using images of serial Z-section (1–1.5 µm). Micrographs of fluorescence microscopy were captured using a BX50 microscope (Olympus, Tokyo, Japan) equipped with a VB7010 digital CCD camera (Keyence, Osaka, Japan). Image processing and movie construction was performed with Adobe Photoshop CS4 and Image J 1.34, respectively.

### Cell culture and transfection

HT1080, HeLa, and COS-7 cells were cultured in Dulbecco's modified Eagle's medium (DMEM) supplemented with 10% fetal calf serum. Transfection for transient expression was performed using FuGENE HD reagent (Roche, Germany) according to the manufacturer's protocol. Stable transfectants were obtained by retrovirus transfection [62]. Plasmids were transfected into Plat-E packaging cells with FuGENE 6 reagent (Roche). Culture supernatant was added to HT1080 ecoR, HeLa ecoR and COS-7 ecoR cells which are stable transfectants of the ecotropic retrovirus receptor, mCAT-1 (gifts from Hiroto Mizushima, Osaka University). Transfected cells were selected by incubation with a combination of 1 µg/ml puromycin, 10 µg/ml blastcidine S, and/or 300 µg/ml zeocin for at least two weeks. Stable transfectants of *C. elegans* genes were named after the transgene that was transduced. T, D, and B refer to *tsp-15*, *doxa-1*, and *bli-3*, respectively (e. g. HT1080^TDB^ cells express *tsp-15*, *doxa-1*, and *bli-3*).

### Antibodies

Both BLI-3 and DOXA-1 rabbit antiserum was prepared by SCRUM Inc. (Tokyo, Japan). BLI-3 rabbit antiserum was raised against keyhole limpet hemocyanin (KLH)-coupled BLI-3 peptides corresponding to residues 254–269 (N1) and a mixture of residues 1232–1245 and 1478–1490 (Cmix). DOXA-1 rabbit antiserum was raised against a mixture of KLH-coupled DOXA-1 peptides corresponding to residues 170–184 and 326–340. Rabbit serum was purified using a peptide-conjugated sepharose column. Anti TSP-15 monoclonal antibody (2C2) was obtained by immunizing rats with HA-tagged TSP-15 protein into footpads. Hybridoma supernatant was purified by ion-exchange chromatography followed by anti-rat IgG-conjugated Sepharose column chromatography. The 2C2 monoclonal antibody is available for immunoprecipitation.

### Western blot, co-immunoprecipitation, and cell surface biotinylation

Cells were harvested and suspended in lysis buffer (20 mM Tris-HCl, 150 mM NaCl, 2 mM EDTA, pH 7.5) with 1% CHAPS. For surface labeling, cells were incubated with 0.2 mg/ml sulfo-NHS-LC-biotin (Thermo Fisher Scientific, Rockford, IL, USA) at 4°C for 30 min and then lysed. For immunoprecipitation and pull-down assays, cleared cell lysates were incubated with anti-FLAG M2 beads (Sigma-Aldrich, St Louis, MO, USA), rat anti-TSP-15 antibody (2C2)-conjugated agarose, or streptavidin agarose beads (Thermo Fisher Scientific). Cleared cell lysate and immunoprecipitates were blotted with mouse anti-Omni (Xpress) (D-8; Santa Cruz Biotechnology, Santa Cruz, CA, USA), mouse anti-FLAG (M2; Sigma-Aldrich), rabbit anti-BLI-3 (N1), rabbit anti-DOXA-1, mouse anti-actin (MAB1501; Millipore, Bedford, MA, USA), or mouse anti-calnexin (AF18; Abcam, Cambridge, UK) antibodies. HRP-conjugated goat anti-rabbit IgG (Zymed, San Francisco, CA, USA), and donkey anti-mouse IgG (Millipore) were used as secondary antibodies. For the immunoblotting of worms, mixed stages of N2, OB129 or OB218 strains were cultured on 150 mm dishes and harvested. Worms were homogenized by sonication and lysed in 1% Triton X-100. Rat anti-TSP-15 or mouse anti-GFP antibody (3E6, Wako chemicals, Osaka, Japan), and anti-rat IgG- or anti-mouse IgG-conjugated Sepharose was added to the cleared worm lysate. Normal rat IgG or mouse IgG was used as a negative control for specific antibodies. Immunoprecipitates were blotted with anti BLI-3 (Cmix) antibody.

### Monitoring H_2_O_2_ production in mammalian cells

The H_2_O_2_ release into culture supernatants was measured using Amplex Red reagent (10-acetyl-3,7-dihydroxyphenoxazine; Invitrogen), which reacts with H_2_O_2_ and is transformed into fluorescent resorufin in the presence of peroxidase [65]. Stable or transient transfectants were plated on 96-well plates at 1–5×10^4^ cells/well, and cultured for 24–48 h. Cells were incubated in 100 µl Hanks' balanced salt solution containing 50 µM Amplex Red, and 0.1 U/ml HRP (Nacalai Tesque, Kyoto, Japan), with or without 10 µM diphenyleneiodonium (DPI), 1 µM ionomycin, 1 µM forskolin (Fsk), or 1 µM phorbol 12-myristate 13-acetate (PMA) at 37°C for 1 h. The fluorescence (530 nm excitation, 590 nm emission) was measured by a Power Scan HT (DS Pharma Biomedicals, Osaka, Japan). Fold-increase was compared with the basal activity of non-transfected HT1080 cells and was determined from least four independent experiments.

### HVJ–mediated cell fusion

HVJ (Sendai virus)-mediated cell fusion was performed using GenomONE-CF (Ishihara Sangyo Co. Ltd., Osaka, Japan). Cells were harvested using trypsin. HT1080^DB^ or HT1080^DBP1311L^ cells (8×10^5^) were mixed with HT1080 ecoR or HT1080^T^ cells (8×10^5^) in a total volume of 200 µl reaction buffer. A 1 µl volume of inactivated HVJ was then added to the cell mixture. After incubation for 15 min at 37°C, 1 ml of DMEM was added and further incubated for 1 h at 37°C for recovery. Cells were treated with 10 µg/ml cycloheximide (Sigma-Aldrich) during incubation for inhibition of protein synthesis. Cells were washed, and H_2_O_2_ production was measured as described above. For the time-course experiment, recovery incubation was examined from 0–120 min, and H_2_O_2_ production was measured after a 30 min incubation. For visualization of the fusion event, HT1080^DB^ cells were pre-stained with 10 µM Cell Tracker Orange (Invitrogen) for 20 min at 37°C, and then fused with GFP-expressing HT1080 cells. Each experiment was tested in duplicate and performed at least three times.

### Statistical analysis

Data are presented as mean value and error bars indicate the standard error of the mean (SEM) from multiple independent assays. Significance was determined using a two-tailed Student's *t*-test.

## Supporting Information

Figure S1 Structure of the *tsp-15* gene, allele, expression and mutant phenotypes. (A) Mutation, deletion locus and most frequent splicing pattern from the *tsp-15* mutant alleles are shown. The *sv15* mutation is a change in the splice donor site of intron 4, as indicated by the arrow. The regions of deletions in *gk201*, *ok854*, *ok881*, and *tm1666* alleles are indicated by bold lines. Three of these deletions, with the exception of *gk201*, result in the lack of most of the second extracellular domain such that these products are no longer functional. In contrast, the *gk201* mutant was indistinguishable from wild type animals (data not shown) despite a 425 bp deletion in the 5′ flanking sequence (−703 to −278) of *tsp-15*, indicating that the deletion sequence does not include an essential element for *tsp-15* transcription. (B) The *tsp-15* hypomorph and null mutant, *sv15* has the Dpy and Bli phenotype. Small blisters are indicated by arrows. Homozygotes of the *tsp-15* deletion alleles show identical recessive embryonic lethal phenotypes. Scanning electron microscopy images show the representative *tsp-15* null mutant, *ok881*. The *ok881* homozygote is short and fat, showing croissant-like morphology and also has a wrinkled cuticle. Scale bars indicate 50 µm in larva and 10 µm in the embryo. (C) Time lapse images of TSP-15::GFP [51] expression patterns and *tsp-15* null embryos during embryogenesis. Upper panels are confocal images of *tsp-15::gfp* expression and Nomarski images of the corresponding embryo. Developmental stages are indicated on the top of the micrographs. *tsp-15::gfp* expression was visible in quadrants of cells along the anteroposterior axis. The expression was decreased and body surface expression was visible around the three-fold stage. TSP-15::GFP expression is prominent in lateral hypodermal cells. Lower panels show representative images of *tsp-15(ok854)* null mutant embryos. Each developmental stage is the same as shown in the upper panels. The *tsp-15(ok854)* embryo developed normally, however, at terminal embryogenesis, *tsp-15(ok854)* embryos shrunk and failed to maintain the vermiform shape. Inset represents a different focal plane, which depicts an abnormal body protrusion in *tsp-15* null embryos. Scale bars indicate 10 µm.
(PDF)

Figure S2 The *bli-3*, *doxa-1* and *mlt-7* mutants, RNAi, and rescue assays. (A) The *bli-3* deletion mutant, *gk141*, is lethal to embryos demonstrating developmental arrest with abnormal body shape at late embryogenesis. The *gk141* mutant was rescued by hypodermal specific expression of *bli-3* cDNA and genomic fragments (a 14.9 kb fragment from fosmid WRM065cD06) containing the *bli-3* gene. Scale bars indicate 50 µm. (B) *doxa-1(RNAi)* animals displayed Bli phenotype as indicated by arrows. The *im21* mutant was rescued by hypodermal specific expression of *doxa-1* cDNA and the Venus-tagged *doxa-1* gene. Scale bars indicate 50 µm. (C) Deficiencies in cuticle development in *mlt-7(rof)* animals. Dumpy phenotype in *mlt-7(im39)* mutants, and moulting defects and blister phenotypes in *mlt-7(RNAi)* animals are shown. The *im39* mutant was rescued by hypodermal-specific expression of *mlt-7*. Arrows depict blisters in *mlt-7(RNAi)* animals, and arrowheads indicate body constriction caused by incomplete shedding of old cuticles during the moulting process. Scale bars indicate 50 µm.
(PDF)

Figure S3 DOXA-1, the homologue of the mammalian DUOXA protein. Multiple alignment of amino acid sequences corresponding to the human DUOXA1 alpha isoform (ACH57453.1), human DUOXA2 (NP_997464.2), and nematode DOXA-1 (NP_498886.2). Identical and similar residues are indicated.
(PDF)

Figure S4 Expression pattern of *doxa-1*. (A) Structure of the *doxa-1::venus* transgene. Boxes indicate exons. (B) Confocal images of the expression pattern of DOXA-1::Venus. *im21* rescued by *doxa-1::venus* is shown. Higher magnification of pharynx, body surface, gonadal arm and vulval regions were also shown. DOXA-1::Venus was expressed in the terminal bulb of the pharynx (tb), hypodermis (especially in seam cells (sc)), distal region of the gonadal arm (g), vulva (v), spermatheca (sp), and uterus (u). Scale bar is indicative of 50 µm.
(PDF)

Figure S5 TSP-15 is highly glycosylated in mammalian cells. Xpress-tagged *tsp-15* or *tsp-15* carrying a mutation in the *N*-glycosylation site (N161Q) was transiently expressed in COS-7 cells. Tunicamycin was added at 0.2 µg/ml for 24 h to partially inhibit *N*-glycosylation. Surface molecules were biotinylated, and cells were lysed with 1% Triton X-100. The lysate was immunoprecipitated with anti-TSP-15 antibody or pull-downed by streptavidin beads. The precipitates were treated with *N*-glycanase (PNGase F; New England Biolabs.) at 37°C for 24 h. Arrows indicate the deglycosylated form of TSP-15.
(PDF)

Figure S6 Association of TSP-15 with DOXA-1. Co-immunoprecipitation of TSP-15 with DOXA-1. BLI-3 was transiently expressed in COS-7 stable transfectants expressing Xpress::TSP-15 and DOXA-1::FLAG or DOXA-1::FLAG alone. A 1% CHAPS cell lysate was used for immunoprecipitation with anti-FLAG antibody. TSP-15 was co-immunoprecipitated with DOXA-1 irrespective of the presence of BLI-3.
(PDF)

Table S1 Strains and mutants used in this study.
(PDF)

Table S2 Isolated mutants similar to *tsp-15(sv15)*.
(PDF)

Text S1 Supplemental Materials and Methods.
(DOC)

Video S1 Time-lapse video microscopy of the *tsp-15* null embryo. Confocal time-lapse imaging of the OB129 *tsp-15(ok854); imEx89[tsp-15p::HisXp::tsp-15]* strain. Nuclear expression of *sur-5::gfp* injection marker depicts rescued or spontaneously array-lost *tsp-15(0)* embryos. Two pairs of *tsp-15(+)* and *tsp-15(0)* embryos are shown. The *tsp-15(0)* (i.e. GFP (−)) embryos were elongated and developed normally, but shrunk at the terminal stages and were unable to maintain a vermiform shape.
(MOV)
