# Supplementary figures and images for: Tetraspanin Is Required for Generation of Reactive Oxygen Species by the Dual Oxidase System in Caenorhabditis elegans
Source: PLoS Genet. 2012 Sep 20;8(9):e1002957. doi: 10.1371/journal.pgen.1002957 (PMC3447965; doi:10.1371/journal.pgen.1002957)

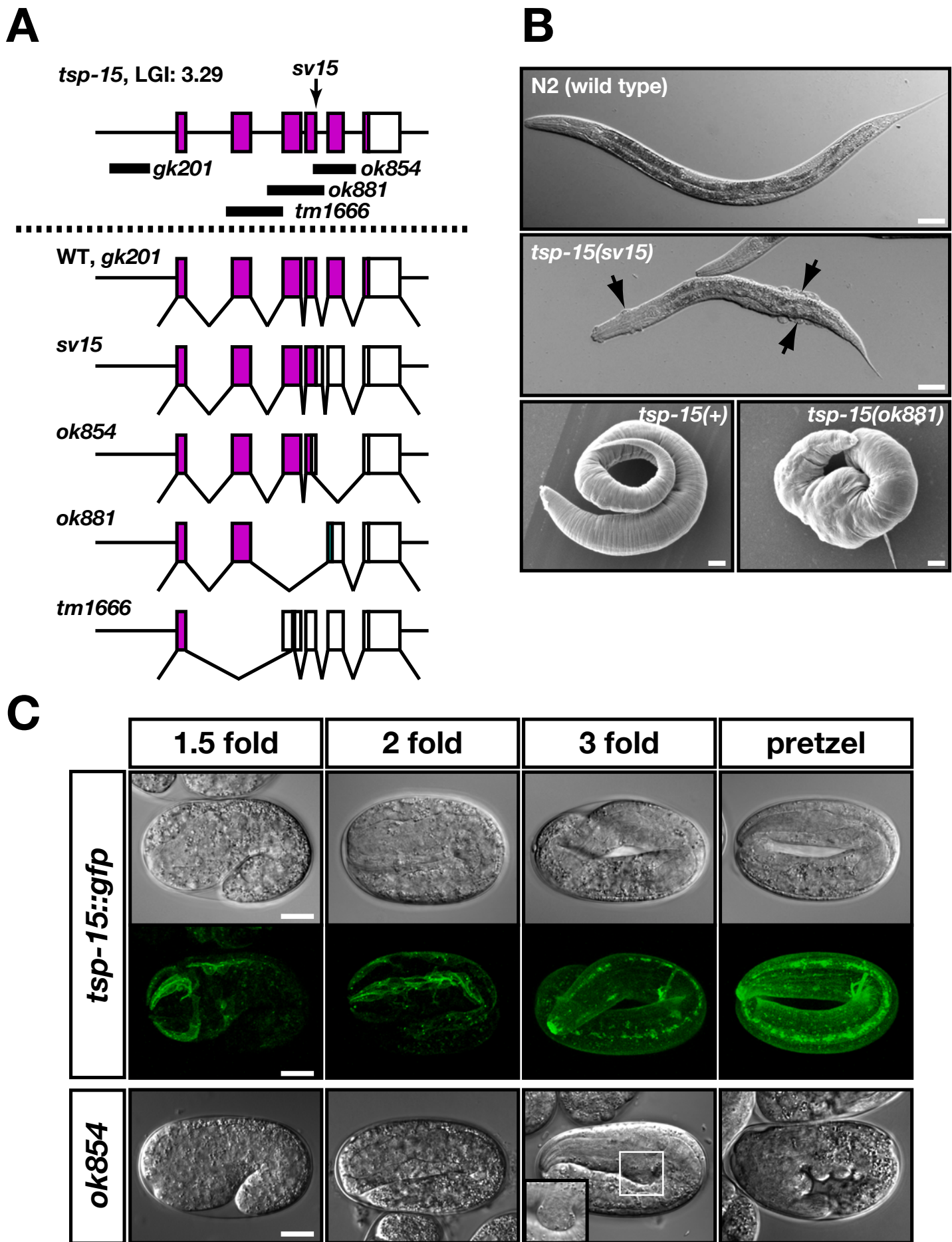

Fig. S1

Supplement: Figure S1 — Structure of the tsp-15 gene, allele, expression and mutant phenotypes. (A) Mutation, deletion locus and most frequent splicing pattern from the tsp-15 mutant alleles are shown. The sv15 mutation is a change in the splice donor site of intron 4, as indicated by the arrow. The regions of deletions in gk201, ok854, ok881, and tm1666 alleles are indicated by bold lines. Three of these deletions, with the exception of gk201, result in the lack of most of the second extracellular domain such that these products are no longer functional. In contrast, the gk201 mutant was indistinguishable from wild type animals (data not shown) despite a 425 bp deletion in the 5′ flanking sequence (−703 to −278) of tsp-15, indicating that the deletion sequence does not include an essential element for tsp-15 transcription. (B) The tsp-15 hypomorph and null mutant, sv15 has the Dpy and Bli phenotype. Small blisters are indicated by arrows. Homozygotes of the tsp-15 deletion alleles show identical recessive embryonic lethal phenotypes. Scanning electron microscopy images show the representative tsp-15 null mutant, ok881. The ok881 homozygote is short and fat, showing croissant-like morphology and also has a wrinkled cuticle. Scale bars indicate 50 µm in larva and 10 µm in the embryo. (C) Time lapse images of TSP-15::GFP [51] expression patterns and tsp-15 null embryos during embryogenesis. Upper panels are confocal images of tsp-15::gfp expression and Nomarski images of the corresponding embryo. Developmental stages are indicated on the top of the micrographs. tsp-15::gfp expression was visible in quadrants of cells along the anteroposterior axis. The expression was decreased and body surface expression was visible around the three-fold stage. TSP-15::GFP expression is prominent in lateral hypodermal cells. Lower panels show representative images of tsp-15(ok854) null mutant embryos. Each developmental stage is the same as shown in the upper panels. The tsp-15(ok854) embryo developed normal [file pgen.1002957.s001.pdf]

**A**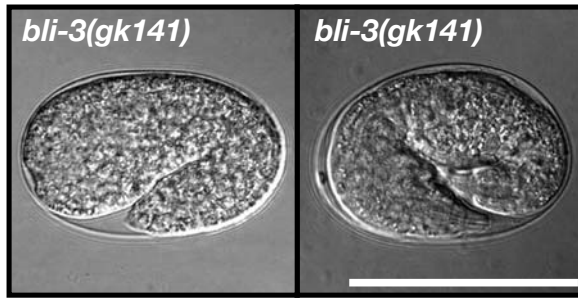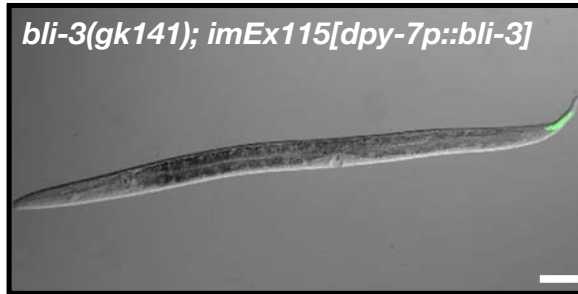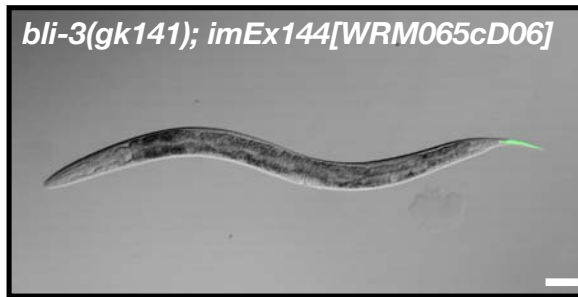**B**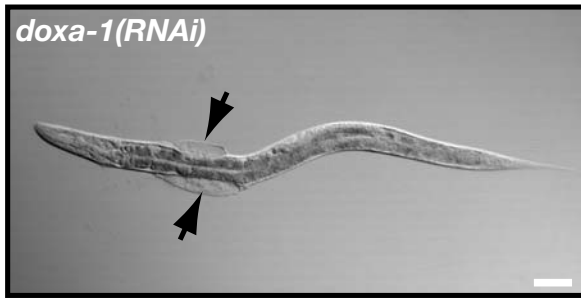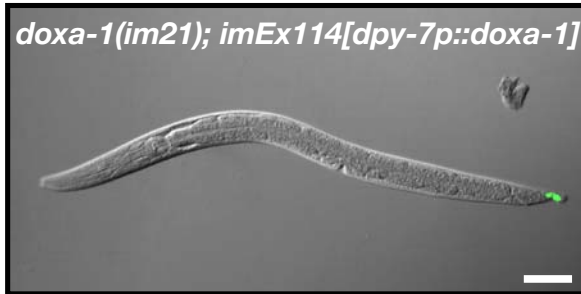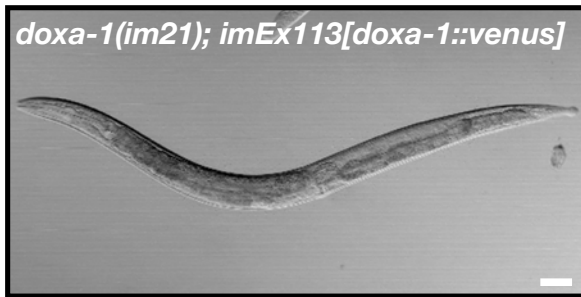**C**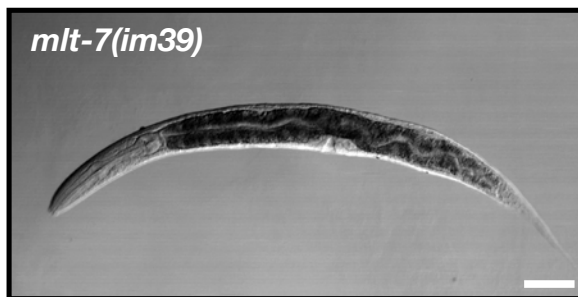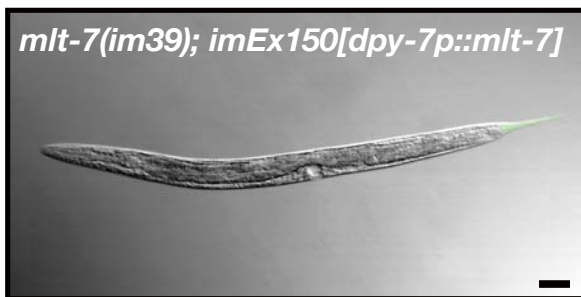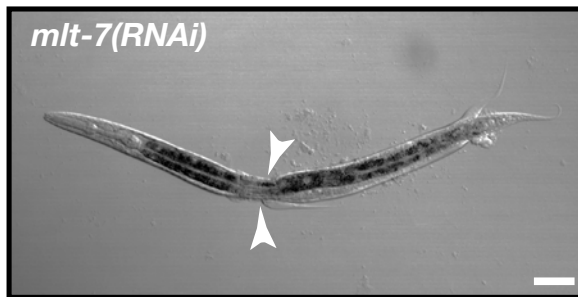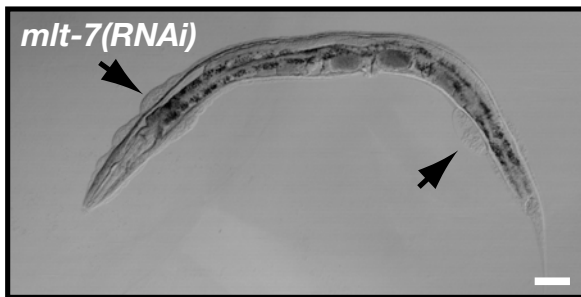**Fig. S2**

Supplement: Figure S2 — The bli-3, doxa-1 and mlt-7 mutants, RNAi, and rescue assays. (A) The bli-3 deletion mutant, gk141, is lethal to embryos demonstrating developmental arrest with abnormal body shape at late embryogenesis. The gk141 mutant was rescued by hypodermal specific expression of bli-3 cDNA and genomic fragments (a 14.9 kb fragment from fosmid WRM065cD06) containing the bli-3 gene. Scale bars indicate 50 µm. (B) doxa-1(RNAi) animals displayed Bli phenotype as indicated by arrows. The im21 mutant was rescued by hypodermal specific expression of doxa-1 cDNA and the Venus-tagged doxa-1 gene. Scale bars indicate 50 µm. (C) Deficiencies in cuticle development in mlt-7(rof) animals. Dumpy phenotype in mlt-7(im39) mutants, and moulting defects and blister phenotypes in mlt-7(RNAi) animals are shown. The im39 mutant was rescued by hypodermal-specific expression of mlt-7. Arrows depict blisters in mlt-7(RNAi) animals, and arrowheads indicate body constriction caused by incomplete shedding of old cuticles during the moulting process. Scale bars indicate 50 µm. (PDF) [file pgen.1002957.s002.pdf]

**A**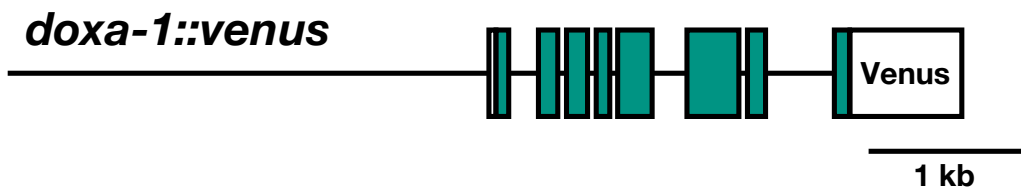**B**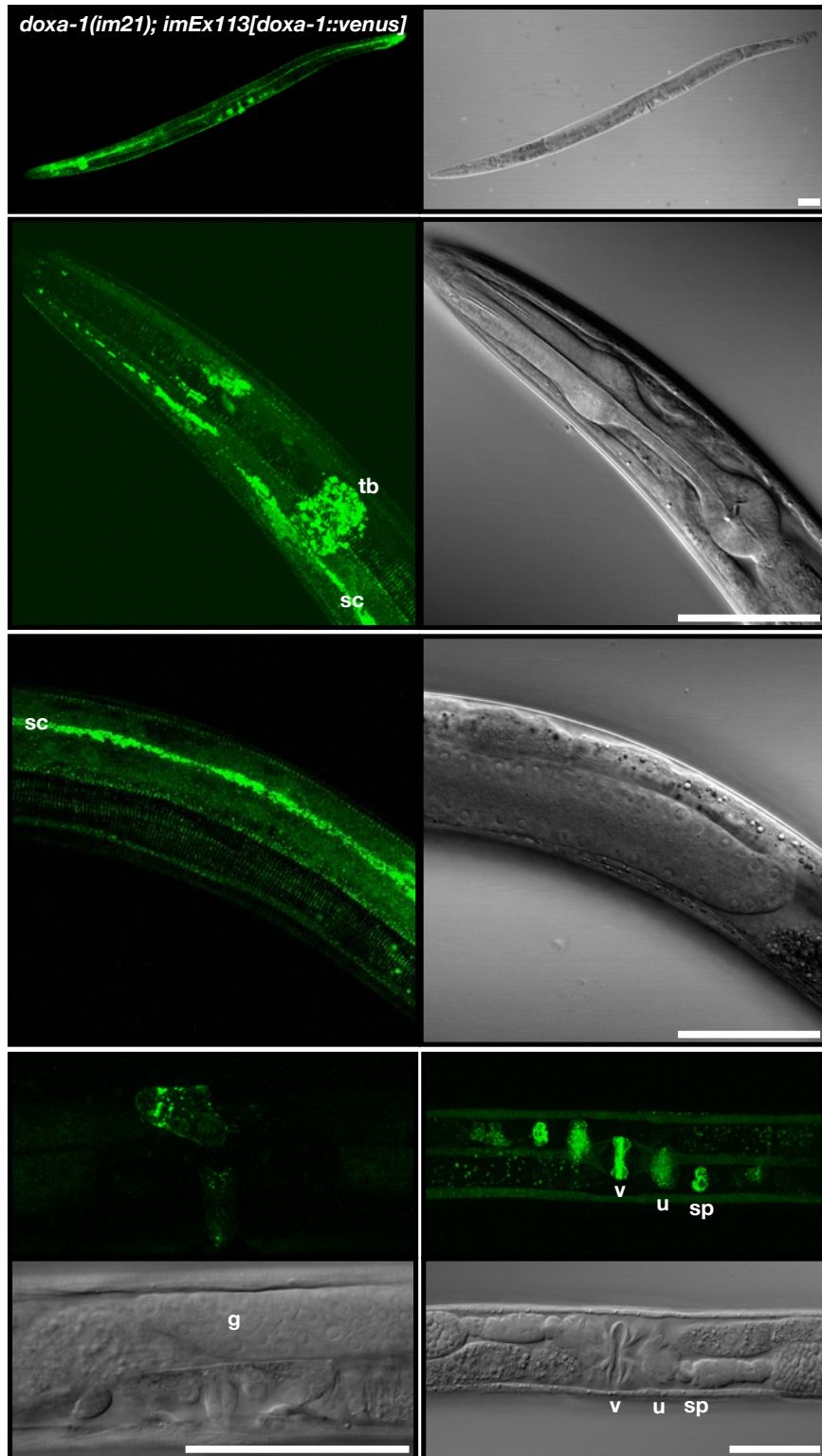**Fig. S4**

Supplement: Figure S4 — Expression pattern of doxa-1. (A) Structure of the doxa-1::venus transgene. Boxes indicate exons. (B) Confocal images of the expression pattern of DOXA-1::Venus. im21 rescued by doxa-1::venus is shown. Higher magnification of pharynx, body surface, gonadal arm and vulval regions were also shown. DOXA-1::Venus was expressed in the terminal bulb of the pharynx (tb), hypodermis (especially in seam cells (sc)), distal region of the gonadal arm (g), vulva (v), spermatheca (sp), and uterus (u). Scale bar is indicative of 50 µm. (PDF) [file pgen.1002957.s004.pdf]

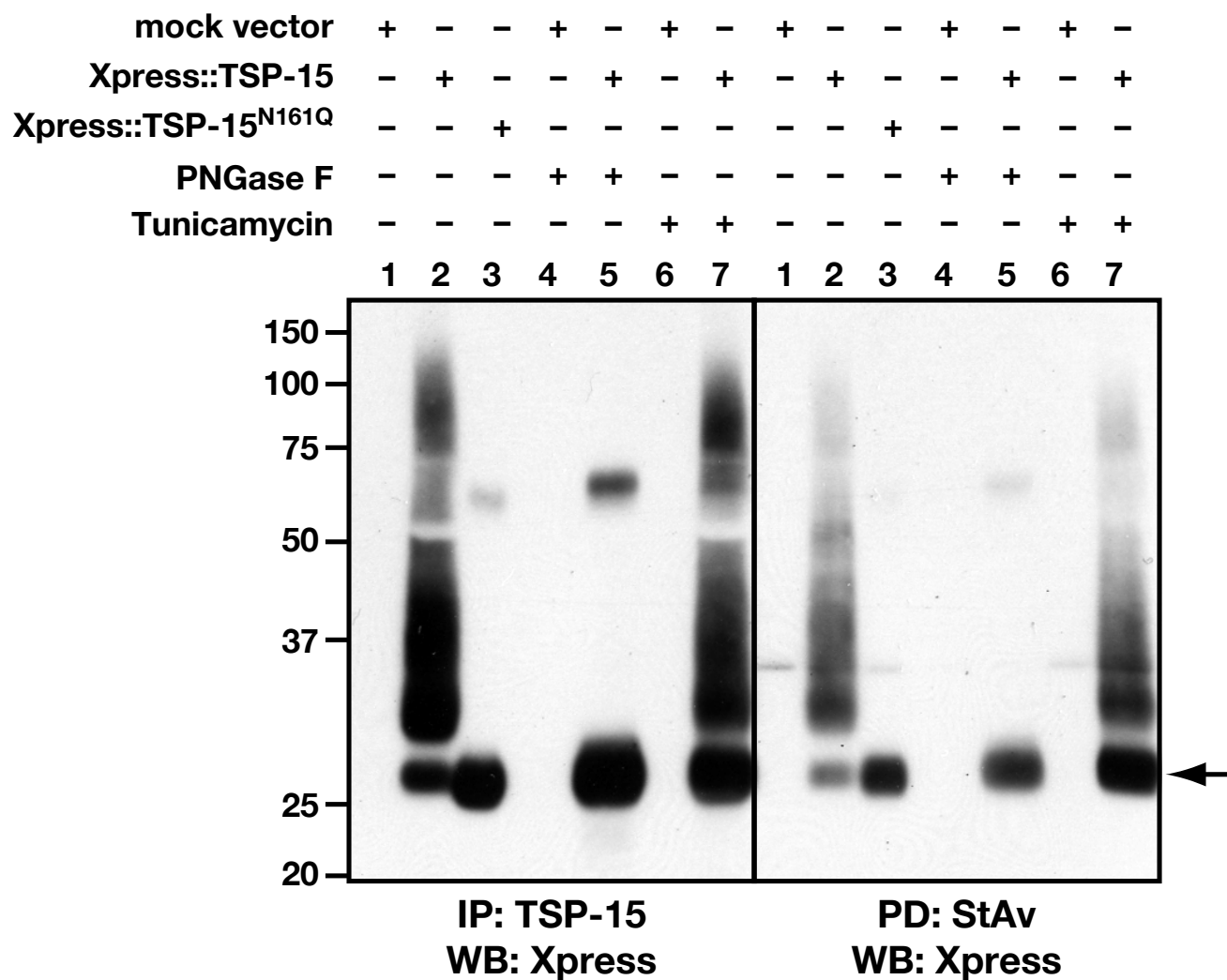

**Fig. S5**

Supplement: Figure S5 — TSP-15 is highly glycosylated in mammalian cells. Xpress-tagged tsp-15 or tsp-15 carrying a mutation in the N-glycosylation site (N161Q) was transiently expressed in COS-7 cells. Tunicamycin was added at 0.2 µg/ml for 24 h to partially inhibit N-glycosylation. Surface molecules were biotinylated, and cells were lysed with 1% Triton X-100. The lysate was immunoprecipitated with anti-TSP-15 antibody or pull-downed by streptavidin beads. The precipitates were treated with N-glycanase (PNGase F; New England Biolabs.) at 37°C for 24 h. Arrows indicate the deglycosylated form of TSP-15. (PDF) [file pgen.1002957.s005.pdf]

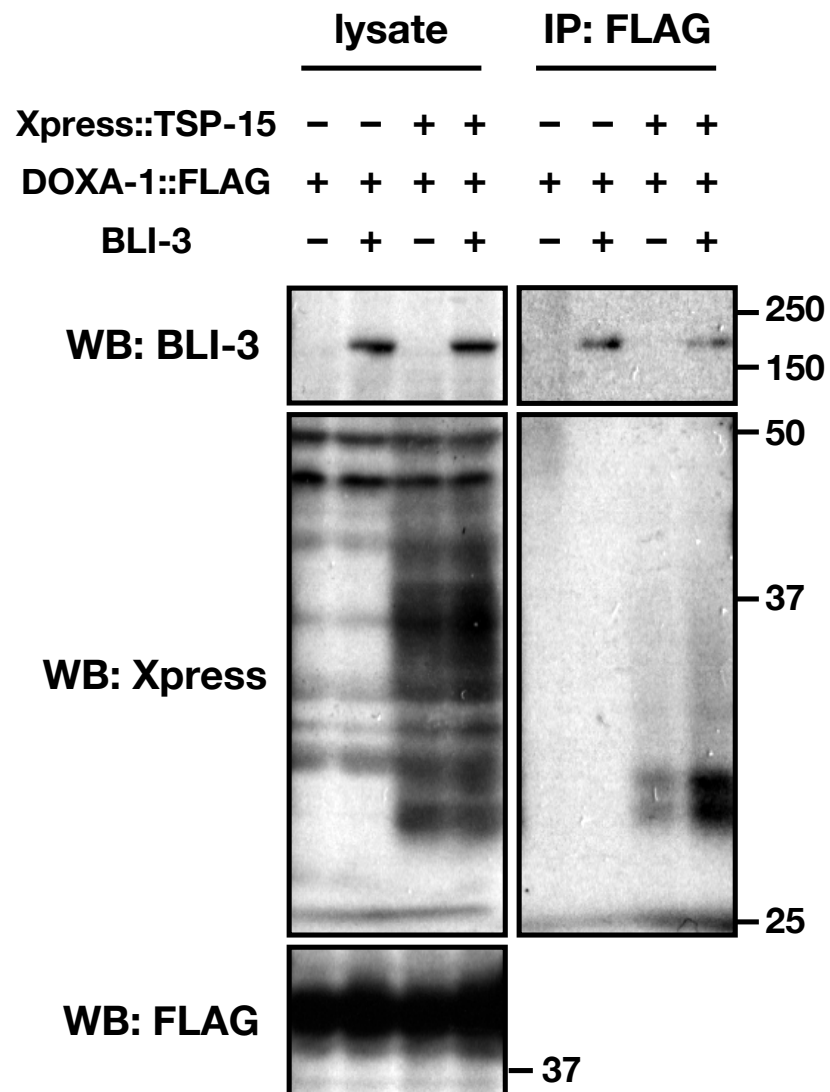

**Fig. S6**

Supplement: Figure S6 — Association of TSP-15 with DOXA-1. Co-immunoprecipitation of TSP-15 with DOXA-1. BLI-3 was transiently expressed in COS-7 stable transfectants expressing Xpress::TSP-15 and DOXA-1::FLAG or DOXA-1::FLAG alone. A 1% CHAPS cell lysate was used for immunoprecipitation with anti-FLAG antibody. TSP-15 was co-immunoprecipitated with DOXA-1 irrespective of the presence of BLI-3. (PDF) [file pgen.1002957.s006.pdf]
