# Supplementary material for: Tetraspanin Is Required for Generation of Reactive Oxygen Species by the Dual Oxidase System in Caenorhabditis elegans
Source: PLoS Genet. 2012 Sep 20;8(9):e1002957. doi: 10.1371/journal.pgen.1002957 (PMC3447965; doi:10.1371/journal.pgen.1002957)
Supplement: Figure S3 — DOXA-1, the homologue of the mammalian DUOXA protein. Multiple alignment of amino acid sequences corresponding to the human DUOXA1 alpha isoform (ACH57453.1), human DUOXA2 (NP_997464.2), and nematode DOXA-1 (NP_498886.2). Identical and similar residues are indicated. (PDF) [file pgen.1002957.s003.pdf]

```
hDUOXA1alpha  MATLGHTFPFYAGPKPTFP-MDTTLASIIMIFLTALATFIVILPGIRGKTRLFWLLRVVT 59
hDUOXA2       MTLWNGVLPFYPQPRHAAG-FSVPLLIVILVFLALAASFLLILPGIRGHSRWFWLVRVLL 59
DOXA-1        MWWFGGNPSPSDYPNAAIPNFNMHAFVIFSVFLIPLIAYILILPGVR-RKRVVTTVTYVL 59
              *   .   .    *. :   :.     :: :**    ::::****:* :.* .  :  :

hDUOXA1alpha  SLFIGAAILAVNFSSEWSVGQVSTNTSYKAFSSEWISADIGLQVGLGGVNITLTGTPVQQ 119
hDUOXA2       SLFIGAEIVAVHFSAEWFVGTVNTNTSYKAFSAARVTARVRLLVGLEGINITLTGTPVHQ 119
DOXA-1        MLAVGGALIASLIYPCWASGSQMIYTQFRGHSNERILAKIGVEIGLQKVNVTLKFERLLS 119
               * :*. ::*  : . *  *     *.::..*   : * : : :**  :*:**.   : .

hDUOXA1alpha  LNETIN--------YNEEFTWRLGENYAEEYAKALEKGLPDPVLYLAEKFTP-RSPCGLY 170
hDUOXA2       LNETID--------YNEQFTWRLKENYAAEYANALEKGLPDPVLYLAEKFTP-SSPCGLY 170
DOXA-1        SNDVLPGSDMTELYYNEGFDISGISSMAEALHHGLENGLPYPMLSVLEYFSLNQDSFDWG 179
               *:.:         *** *     .. *    :.**:*** *:* : * *:    .. .

hDUOXA1alpha  RQYRLAGHYTSAMLWVAFLCWLLANVMLSMPVLVYGGYMLLATGIFQLLALLFFSMATSL 230
hDUOXA2       HQYHLAGHYASATLWVAFCFWLLSNVLLSTPAPLYGGLALLTTGAFALFGVFALASISSV 230
DOXA-1        RHYRVAGHYTHAAIWFAFACWCLS-VVLMLFLPHNAYKSILATGISCLIACLVYLLLSPC 238
              ::*::****: * :*.**  * *: *:*       .   :*:**   *:. :     :.

hDUOXA1alpha  TSPCPLH---LGASVLHTHHGPAFWITLTTGLLCVLLGLAMAVAHRMQPHRLKAFFNQSV 287
hDUOXA2       P-LCPLR---LGSSALTTQYGAAFWVTLATGVLCLFLGGAVVSLQYVRPSALRTLLDQSA 286
DOXA-1        ELRIAFTGENFERVDLTATFSFCFYLIFAIGILCVLCGLGLGICEHWRIYTLSTFLDASL 298
                    .:    :    * : .. .*:: :: *:**:: * .:   .  :   * :::: *

hDUOXA1alpha  DEDPMLEWSPEEGGLLSPRYRSMADS----------------------------------P 314
hDUOXA2       KD-----CSQERGG--SP---LILGD----------------------------------P 303
DOXA-1        DEHVGPKWKKLPTGGPALQGVQIGAYGTNTTNSSRDKNDISSDKTAGSSGFQSRTSTCQS 358
              .:      .    *  :     :                                    .

hDUOXA1alpha  KSQDIPLSEASSTKAYCKEAHPKDPDCAL---------- 343
hDUOXA2       LHKQAALPDLKCITTNL---------------------- 320
DOXA-1        SASSASLRSQSSIETVHDEAELERTHVHFLQEPCSSSST 397
                .. .* . ..  :
```

Fig. S3
