# Supplementary material for: Tetraspanin Is Required for Generation of Reactive Oxygen Species by the Dual Oxidase System in Caenorhabditis elegans
Source: PLoS Genet. 2012 Sep 20;8(9):e1002957. doi: 10.1371/journal.pgen.1002957 (PMC3447965; doi:10.1371/journal.pgen.1002957)
Supplement: Table S1 — Strains and mutants used in this study. (PDF) [file pgen.1002957.s007.pdf]

Table S1. Strains and mutants used in this study.

Strain	Genotype
Bristol N2	wild type
CB4856	Hawaiian variant for SNP source
SP2275	*tsp-15(sv15)*
VC332	*tsp-15(gk201)*
VC633	*tsp-15(ok854)/hT2*
VC634	*tsp-15(ok881)/hT2*
OB69	*tsp-15(tm1666)/hT2*
CB767	*bli-3(e767)*
MT1141	*bli-3(n529)*
OB13	*tsp-15(sv15); imEx13[dpy-7p::HisXp::tsp-15]*
OB14	*tsp-15(sv15); imEx14[tsp-15::gfp]*
OB43	*imIs1[dpy-7p::gfp]*
OB65	*tsp-15(ok854); imEx64[dpy-7p::HisXp::tsp-15]*
OB67	*tsp-15(ok881); imEx65[dpy-7p::HisXp::tsp-15]*
OB70	*tsp-15(tm1666); imEx67[dpy-7p::HisXp::tsp-15]*
OB104	*tsp-15(ok881)/unc-29(e1072) lin-11(n566)*
OB129	*tsp-15(ok854); imEx89[tsp-15p::HisXp::tsp-15]*
OB140	*bli-3(im10)*
OB143	*bli-3(gk141)/hT2*
OB149	*bli-3(im10)/hT2*
OB154	*bli-3(e767); imEx13[dpy-7p::HisXp::tsp-15]*
OB156	*bli-3(n529); imEx13[dpy-7p::HisXp::tsp-15]*
OB208	*doxa-1(im21)/hT2*
OB218	*doxa-1(im21); imEx113[doxa-1::venus]*
OB219	*doxa-1(im21); imEx114[dpy-7p::doxa-1]*
OB220	*bli-3(gk141); imEx115[dpy-7p::bli-3]*
OB247	*tsp-15(sv15); imEx113[doxa-1::venus]*
OB254	*bli-3(gk141); imEx144[bli-3]*
OB257	*tsp-15(sv15); imEx147[bli-3, doxa-1::venus]*
OB262	*tsp-15(sv15); imEx144[bli-3]*
OB263	*tsp-15(ok881)/hT2; imEx147[bli-3, doxa-1::venus]*
OB265	*bli-3(e767); imEx144[bli-3]*
OB266	*bli-3(im10); imEx144[bli-3]*
OB267	*bli-3(e767); imEx113 [doxa-1::venus]*
OB270	*bli-3(e767); imEx148[dpy-7p::HisXp::tsp-15, doxa-1::venus]*

OB271	*doxa-1(im21); imEx149[dpy-7p::HisXp::tsp-15, bli-3]*

Strains were obtained from the *Caenorhabditis* Genetic Centre, the *C. elegans* Gene Knockout Consortium, and National BioResouce Project Japan. Descriptions of the strains are available on Wormbase (http://www.wormbase.org). OB strains and their information can be provided upon request.
