# Supplementary material for: Tetraspanin Is Required for Generation of Reactive Oxygen Species by the Dual Oxidase System in Caenorhabditis elegans
Source: PLoS Genet. 2012 Sep 20;8(9):e1002957. doi: 10.1371/journal.pgen.1002957 (PMC3447965; doi:10.1371/journal.pgen.1002957)
Supplement: Table S2 — Isolated mutants similar to tsp-15(sv15). (PDF) [file pgen.1002957.s008.pdf]

Table S2. Isolation of mutants similar to *tsp-15(sv15)*.

allele	CG	sequence	gene	mutation
im10	1	F56C11.1	*bli-3*	missense (P1311L)
im11	2			
im12	2			
im13	1			
im14	2			
im16	2			
im20	1			
im21	3	C06E1.3	*doxa-1*	splice error
im26	1			
im27	2			
im32	3	C06E1.3	*doxa-1*	splice error
im38	4	ZK480.3	*mlt-7*	missense (F375S)
im39	4	ZK480.3	*mlt-7*	missense (I343S)

CG, complementation group.
