# Supplementary material for: Tetraspanin Is Required for Generation of Reactive Oxygen Species by the Dual Oxidase System in Caenorhabditis elegans
Source: PLoS Genet. 2012 Sep 20;8(9):e1002957. doi: 10.1371/journal.pgen.1002957 (PMC3447965; doi:10.1371/journal.pgen.1002957)
Supplement: Text S1 — Supplemental Materials and Methods. (DOC) [file pgen.1002957.s009.doc]

**Text S1.**

**Supplemental Materials and Methods**

RNA interference

RNAi by bacterial feeding was performed as described previously [1]. The *tsp-15*, *bli-3*, *doxa-1* and *mlt-7* cDNA fragments were cloned into pPD129.36 and transformed into *HT115(DE3)* *E. coli*. Transformants were spotted onto NGM agar plates supplemented with 1 mM IPTG and 100 μg/ml ampicillin. IPTG induction was performed overnight at room temperature. Late L4 larvae were fed on the plate and transferred at 12 hours intervals over three days, and the F1 phenotype was assayed.

DNA transformation of worms

Microinjection of worms for DNA transformation was performed as described previously [2]. Transgenes were injected at a concentration of 1–50 ng/μl. The *lin-44::gfp* or *sur-5::gfp* constructs at 25–50 ng/μl were used as a co-injection marker. The total amount of injected DNA was 100 ng/μl with pBSSK. Phenotypes were assayed using three independent transgenic lines.

Scanning electron microscopy (SEM)

Eggs laid on a plate from OB104 were scraped off gently with a rubber policeman. Eggs were pre-treated with alkaline hypochlorite, then eggshells were digested with 15 mg/ml Yatalase™ (Takara Bio, Japan) in egg buffer (118 mM NaCl, 48 mM KCl, 2 mM CaCl_2_, 2 mM MgCl_2_, 25 mM HEPES, pH7.4). Vitelline membrane was removed by gentle pipetting, and embryos were fixed in 1% glutaraldehyde in 0.1 M sodium phosphate buffer (pH 7.2). Embryos were mounted on MAS-coated slide glasses (Matsunami glass, Japan) and conductive stained with 1% tannic acid and 1% osmium tetroxide. After washing with sodium phosphate buffer, samples were dehydrated through a graded series of ethanol, and dried in a critical point dryer (CPD-2, Hitachi, Japan) using liquid CO_2_. The dried samples were coated with platinum/palladium in an ion coater and observed with an S-4300SE/N scanning electron microscope (Hitachi, Japan).

**Supplemental References**

1. Kamath RS, Martinez-Campos M, Zipperlen P, Fraser AG, Ahringer J (2001) Effectiveness of specific RNA-mediated interference through ingested double-stranded RNA in *Caenorhabditis elegans*. Genome Biol 2: RESEARCH0002.0001-0010.

2. Mello C, Fire A (1995) DNA transformation. Methods Cell Biol 48: 451-482.
